# MAPK-triggered chromatin reprogramming by histone deacetylase in plant innate immunity

**DOI:** 10.1186/s13059-017-1261-8

**Published:** 2017-07-06

**Authors:** David Latrasse, Teddy Jégu, Huchen Li, Axel de Zelicourt, Cécile Raynaud, Stéphanie Legras, Andrea Gust, Olga Samajova, Alaguraj Veluchamy, Naganand Rayapuram, Juan Sebastian Ramirez-Prado, Olga Kulikova, Jean Colcombet, Jean Bigeard, Baptiste Genot, Ton Bisseling, Moussa Benhamed, Heribert Hirt

**Affiliations:** 1 Institute of Plant Sciences Paris-Saclay (IPS2), CNRS, INRA, University Paris-Sud, University of Evry, University Paris-Diderot, Sorbonne Paris-Cite, University of Paris-Saclay, Batiment 630, 91405 Orsay, France; 2 Laboratory of Molecular Biology, Wageningen University, Droevendaalsesteeg 1, 6708PB Wageningen, The Netherlands; 3 Division of Biological and Environmental Sciences and Engineering, King Abdullah University of Science and Technology, Thuwal, 23955-6900 Saudi Arabia; 4 Plateforme Biopuces et séquençage, IGBMC, 1 rue Laurent Fries Parc d’Innovation, 67400 Illkirch, France; 5 Center for Plant Molecular Biology, University of Tübingen, Auf der Morgenstelle 32, 72076 Tübingen, Germany; 6 Centre of the Region Haná for Biotechnological and Agricultural Research, Faculty of Science, Palacký University Olomouc, Šlechtitelů 27, 783 71 Olomouc, Czech Republic

## Abstract

**Background:**

Microbial-associated molecular patterns activate several MAP kinases, which are major regulators of the innate immune response in *Arabidopsis thaliana* that induce large-scale changes in gene expression. Here, we determine whether microbial-associated molecular pattern-triggered gene expression involves modifications at the chromatin level.

**Results:**

Histone acetylation and deacetylation are major regulators of microbial-associated molecular pattern-triggered gene expression and implicate the histone deacetylase HD2B in the reprogramming of defence gene expression and innate immunity. The MAP kinase MPK3 directly interacts with and phosphorylates HD2B, thereby regulating the intra-nuclear compartmentalization and function of the histone deacetylase.

**Conclusions:**

By studying a number of gene loci that undergo microbial-associated molecular pattern-dependent activation or repression, our data reveal a mechanistic model for how protein kinase signaling directly impacts chromatin reprogramming in plant defense.

**Electronic supplementary material:**

The online version of this article (doi:10.1186/s13059-017-1261-8) contains supplementary material, which is available to authorized users.

## Background

Due to their sessile nature, plants have developed sophisticated ways to respond and adapt to a variety of external stress factors that would otherwise compromise proper development, reproductive success, and ultimately survival. Numerous cellular proteins interact and communicate in response to extracellular stimuli and, through multiple signaling networks, transmit signals to the nucleus for reprogramming chromosomal gene expression. These dynamic regulatory mechanisms contribute to the capacity of plants to adapt to the onslaught of both biotic and abiotic challenges. Indeed, the success of photosynthetic eukaryotes is influenced by the adaptive dynamics of chromatin regulatory mechanisms, like histone modifications, which are rapid and reversible.

The choice of gene expression ultimately determines the fate of cells, forming the basis of biological diversity. The regulation of gene expression is closely coupled to chromatin structure and its modifications, which determine the accessibility of many regulatory proteins and non-coding RNAs (ncRNAs) to the DNA, adding a further layer of complexity to the genetic information encoded by the DNA sequence [1–3]. Chromatin is a tightly contained higher order structure that compacts genomic DNA to fit within the nucleus. The fundamental unit of chromatin is the nucleosome, which is composed of DNA that is wrapped around an octamer of histone proteins. Chromatin structure is modulated by a variety of mechanisms including DNA methylation catalyzed by DNA cytosine methyltransferases, histone post-translational modifications, such as acetylation and methylation, catalyzed by a wide range of enzymes specific for each modification, alterations in histone-DNA interactions that facilitate nucleosome sliding and are catalyzed by chromatin remodeling complexes, histone variants, and long and small ncRNAs that can act directly on chromatin and induce RNA-dependent DNA methylation (RdDM) [4–6].

Many developmental and environmental cues induce changes in chromatin structure. Plants sense pathogens through the perception of MAMPs, which induce signaling cascades to activate transcription factors and invoke chromatin regulatory mechanisms to reorganize the chromatin structure and, ultimately, provoke the changes in gene expression necessary for plant defense. Thus, in response to different stimuli, a single eukaryotic genome (DNA sequence) can give rise to distinct epigenomes.

Here, we examine the role of chromatin remodeling in *Arabidopsis thaliana* upon challenge with a synthetically produced 22 amino-acid long flagellin peptide (flg22) that mimics the response to bacterial pathogens. Flg22 is recognized in Arabidopsis by the plasma membrane leucine-rich repeat-receptor kinase (LRR-RK) FLS2 and activates two MAPK signaling pathways that initiate an array of defense responses including the production of several hormones, reactive oxygen species, and the induction of a large set of defense genes, processes generally referred to as MAMP-triggered immunity (MTI). These two main cascade branches involve the successive recruitment of MAP kinase kinase kinases (MAP3Ks), which phosphorylate and activate MAP kinase kinases (MAP2Ks) that phosphorylate and activate the MAP kinases MPK3, 4, 6, and 11 [7–9]. Ultimately, these MAPKs phosphorylate and thereby regulate protein factors responsible for the increased or decreased expression of specific gene sets with the goal to counteract the pathogen assault. Although specific transcription factors have been identified as targets for MPK3 [10], MPK4 [11], and MPK6 [12], much of the protein machinery orchestrating gene regulation in response to flagellin has not been identified.

Here, we report a role for the MAP kinase MPK3 in chromatin modulation and dissect the defense mechanism during the response to flagellin. We show that MPK3 interacts directly with and phosphorylates the histone deacetylase HD2B, which has been shown to deacetylate the lysine 9 residue of histone 3 (H3K9), a modification generally linked to the compaction of chromatin. We also show that, upon flagellin perception, HD2B is relocalized from the nucleolus to the nuclear compartment, leading to global genome-wide shifts in the H3K9 acetylation landscape. We identified flg22-regulated defense genes targeted by the MPK3-HD2B regulatory module and show that HD2B is directly implicated in bacterial defense in plants. Our results mechanistically define how a MAMP-activated MAP kinase regulates global changes in the chromatin landscape.

## Results

### MPK3 interacts with and phosphorylates HD2B in vivo

Our previous phosphoproteomic approach aimed at identifying MAPK substrates revealed 303 in vivo phosphorylation sites in proteins isolated from Arabidopsis root cells [13]. Among these sites, 91 matched the proline-directed motifs pS/pT-P that commonly serve as phosphorylation sites for MAPKs. Because we were interested to identify global regulators of gene expression that play a role in pathogen defense, we selected the histone deacetylase (HDAC) HD2B, which was phosphorylated at the amino acid positions T249 and S266, matching the S/T-P consensus motif for MAPK substrates (Additional file 1: Figures S1–S5).

**Fig. 1:**
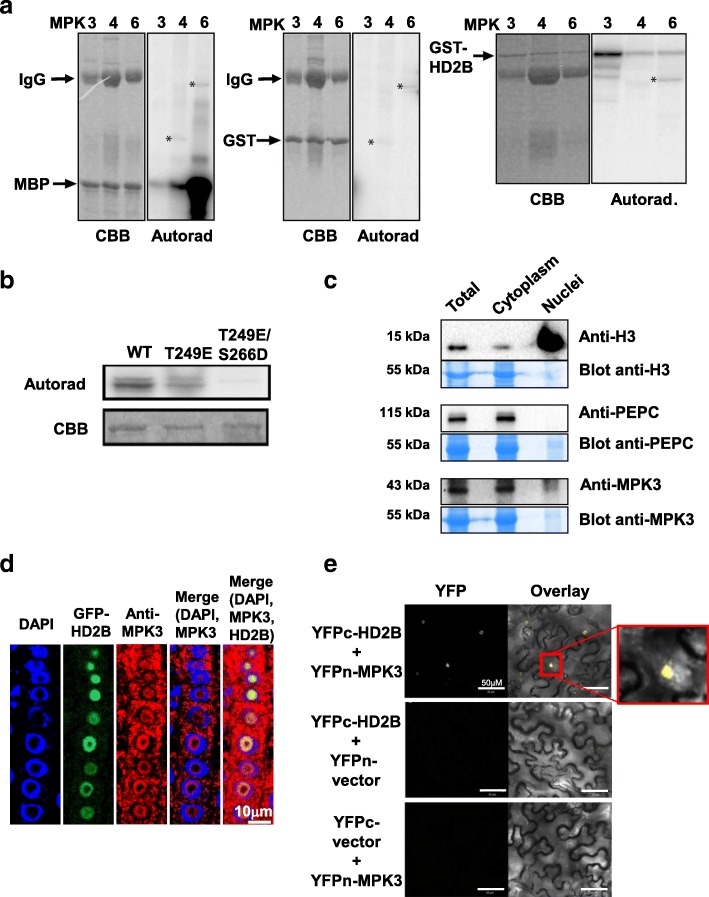
MPK3 phosphorylates HD2B in vitro and interacts with HD2B. **a** In vitro phosphorylation assays of MBP (*left*), GST (*middle*), and HD2B-GST (*right*) by MPK3 (3), MPK4 (4), and MPK6 (6). MPK3, 4, and 6 were immunoprecipitated from flagellin-treated root cell suspensions. For each assay, Coomassie staining of the gel (CBB) is shown on the *left* as a protein loading control and the autoradiography (Autorad) is shown on the *right*. MPK3, 4, and 6 were able to phosphorylate MBP, but not GST. HD2B phosphorylation was observed with all three MPKs, but MPK3 showed the highest activity. Arrows indicate full-length forms of all substrate proteins. IgG indicates immunoglobulin G used for immunoprecipitation of the MAPKs. *Asterisks* indicate an unknown phosphoprotein in the MPK4 lanes, or MPK6 autophosphorylation in the MPK6 lanes. **b** In vitro phosphorylation assays of WT HD2B (WT) and the mutant HD2B protein forms HD2B-T249E and HD2B-T249E/S266D. Coomassie staining of the gel (CBB) is shown as a protein loading control. **c** Cell fractionation assay of MPK3 localization. Proteins from total, cytoplasmic, and nuclear cell extracts were subjected to SDS-PAGE and analyzed by immunoblotting with anti-MPK3 antibody. Anti-H3 and anti-PEPC antibodies were used as controls for nuclear and cytoplasmic proteins, respectively. **d** Immunostaining of MPK3 and HD2B performed on plants expressing a GFP-HD2B fusion. Antibodies used to reveal the presence of the proteins are noted above the panels. DAPI staining was used to label the nuclei. MPK3 could be detected both in the cytoplasm and in the nucleus while GFP-HD2B was only observed in the nucleus. **e** BiFC analysis of HD2B interaction with MPK3 in epidermal cells of *Agrobacterium*-infiltrated *Nicotiana benthamiana*. Empty vectors were used as controls. Fluorescence indicates that yellow fluorescent protein C terminal fragment (YFPc)-HD2B interacts with YFPn-MPK3. YFPc-HD2B does not interact with empty YFPn vector and YFPn-MPK3 does not interact with empty YFPc vector Overlay indicates merging the yellow fluorescent protein (YFP) and light transmission images

We first assayed whether HD2B was a substrate of the canonical MAPK pathways activated by pathogen recognition. To this end, we immuno-purified endogenous flg22-activated MPK3, MPK4, and MPK6 from a root cell culture extract and tested whether the MAPKs phosphorylated HD2B in vitro. Using MBP and GST as positive and negative controls, respectively, MPK3 showed the highest phosphorylation activity toward GST-tagged HD2B among the three tested MPKs (Fig. 1a), whereas MPK4 and MPK6 showed a weaker substrate preference to HD2B when compared with the artificial substrate MBP (Fig. 1a). To control our MAPK antibody specificity, we performed kinase assays with protein extracts from *mpk3*, *mpk4*, *mpk6*, and *mpk7* mutants and their respective wild-type (WT) lines after immunoprecipitation with the corresponding antibodies (Additional file 1: Figure S6). MPK7 antibody was used as a negative control as this kinase is not activated upon flg22 treatment. The results indicated that the antibodies specifically immunoprecipitated the respective kinases (Additional file 1: Figure S6). To confirm the substrate specificity of the phosphorylation of HD2B by MPK3, we also tested several additional substrates that were identified in the phosphoproteome screen. As shown in Additional file 1: Figure S7, PI-4Kß1 (At5g64070) was specifically phosphorylated by MPK3 and MPK6, SCF (At5g13300) was not phosphorylated by any of the three immune MAPKs, MSL9 (At5g19520) and GOS12 (At2g45200) were preferentially phosphorylated by MPK6. To determine whether MPK3 phosphorylated the previously identified HD2B in vivo sites T249 and S266 [13], the T249 amino acid residue was mutated individually to glutamate or in combination with S266 to aspartate residues (HD2B-T249E and HD2B-T249E/S266D, respectively; denominated further as HD2B-ED), and these mutant HD2B proteins together with WT HD2B were tested for their ability to serve as MPK3 substrates (Fig. 1b). In comparison to WT HD2B, HD2B-T249E showed reduced and HD2B-T249E/S266D complete lack of phosphorylation by MPK3, indicating that the in vivo phosphorylation sites T249 and S266 can be targeted by MPK3 in vitro (Fig. 1b).

To better understand where the MPK3 and HD2B interaction takes place, we investigated the subcellular localization of the HD2B and MPK3 proteins. A GFP-HD2B fusion protein was localized in the nucleolus [14, 15], whereas MPK3 was observed in both cytoplasmic and nuclear cell compartments (Fig. 1c and Additional file 1: Figure S8). Using indirect immunofluorescence staining, we found that MPK3 and HD2B were co-localized in the nucleoli of the same plant tissue (Fig. 1d). To determine if MPK3 and HD2B can interact in vivo, we performed a bimolecular fluorescence complementation assay (BiFC). To this end, the MPK3 and HD2B complementary DNAs (cDNAs) were inserted into binary vectors, containing the split yellow fluorescent protein (YFP) N-terminal fragment (YFPn) and the C-terminal fragment (YFPc), respectively. When YFPc-HD2B and YFPn-HD2B were expressed together in transiently transformed *Nicotiana benthamiana* leaves, YFP fluorescence was observed inside nuclei revealing an *in planta* interaction between the two proteins (Fig. 1e, upper panels). No fluorescence was observed when each tagged protein was expressed separately with the corresponding empty vector (Fig. 1e, middle and bottom panels). Because both MPK4 and MPK6 showed a capacity to phosphorylate HD2B, but with a weaker preference than MPK3, we tested their interaction with HD2B by BiFC. As expected we found that MPK4 and MPK6 can also interact with HD2B but with a lower efficiency (Additional file 1: Figure S9). Due to the fact that MPK3 showed the highest phosphorylation activity we decided to focus our study on this specific MAPK. To confirm the MPK3/HD2B interaction in vivo we performed co-immunoprecipitation experiments. First, protein extracts obtained from protoplasts transiently expressing *HD2B-c-Myc* and *MPK3-HA* tagged proteins were immunoprecipitated with an anti-c-Myc antibody and analyzed by immunoblotting with anti-c-Myc or anti-HA antibodies. We observed that HD2B interacts with MPK3 (Additional file 1: Figure S10A) consistent with our in vitro kinase assays (Fig. 1a). Second, co-IP assays performed with transgenic plants expressing both *GFP-HD2B* and *MPK3-c-Myc* tagged proteins (Additional file 1: Figure S10B) confirmed our BiFC assays (Fig. 1e) that HD2B interacts with MPK3 in vivo. Taken together, our results showed that MPK3 interacts with and phosphorylates HD2B.

### The MPK3-HD2B module controls the transcription of biotic stress response genes by modulating H3K9ac levels

**Fig. 2:**
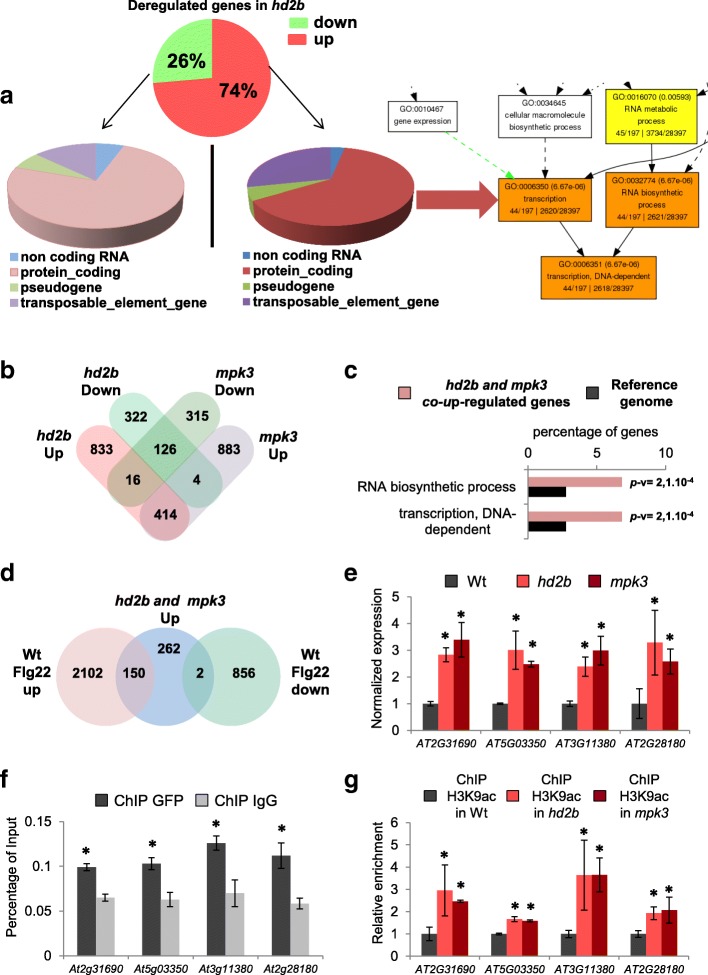
HD2B and MPK3 regulate the basal expression of a subset of defense genes. **a** Annotation of *hd2b* deregulated genes. Twenty-six percent of *hd2b* deregulated genes are downregulated (*green*, 452 genes) whereas 74% are upregulated (*red*, 1263 genes). Generation of a hierarchical tree graph with the Agrigo GO Analysis Toolkit shows that 63% of the upregulated genes code for proteins with significant enrichment in “transcription” and “RNA biosynthetic process.” **b** Comparisons among the transcriptomes of *hd2b* and *mpk3* mutants. Thirty-two percent of upregulated (414) and 29% of downregulated (126) genes in mpk3 are in common with *hd2b* mutants. **c** GO analysis of commonly upregulated genes in *hd2b* and *mpk3* mutants with the Agrigo GO Analysis Toolkit. Histograms of the values highlight the enrichment of genes involving RNA biosynthesis and transcription. *P* values for each enriched class are indicated (*p*-v). **d** Comparisons of the 414 commonly upregulated genes in *hd2b* and *mpk3* mutants with the genes upregulated or downregulated in WT seedlings by flg22 treatment for 30 min. **e** Validation of transcriptomic data by quantitative reverse transcription polymerase chain reaction (RT-qPCR). The upregulation of four genes (of the 150 commonly upregulated genes in *hd2b* and *mpk3* and flg22-treated WT in Fig. 2d) was confirmed by RT-qPCR. **f** HD2B protein binding to flg22-inducible genes in mock conditions. Chromatin immunoprecipitation (ChIP)-qPCR assays with anti-GFP antibodies were performed on *pHD2B::GFP-HD2B* seedlings (Fig. 2e). An IgG antibody was used as a negative control. **g** MPK3 and HD2B promote H3K9 deacetylation of flg22-inducible genes in mock conditions. Using an anti-H3K9ac antibody, ChIP-qPCR assays were performed in *hd2b*, *mpk3*, and WT seedlings. Compared with WT, the four loci (Fig. 3e and f) were hyperacetylated in *hd2b* and *mpk3* mutants

To gain insight into the role of HD2B and its interaction with MPK3 in gene regulation and plant defense, we examined the gene expression consequences of HD2B and MPK3 loss-of-function mutants in resting conditions and in flagellin-challenged plants. To this end, we performed RNA-sequencing (RNA-seq) analyses on *hd2b* and *mpk3* mutants and WT plants (Additional files 2 and 3: Tables SI and SII) either mock-treated for 30 min or treated for the same period of time with 1 μM of flg22. First, when compared to WT, we observed that 1714 genes were deregulated in *hd2b* with a fold change > 2 and *p* value < 0.05. From these 1714 genes, 74% were upregulated and 26% downregulated (Fig. 2a). This pattern is consistent with the role of HD2B as a repressor. A functional annotation of *hd2b* upregulated genes under control conditions showed a clear enrichment in genes involved in transcription (Fig. 2a and Additional file 1: Figure S11). By contrast, an annotation of downregulated genes showed enrichment in genes involved in transport activity (Additional file 1: Figure S12A and S12B). Because HD2B is a histone deacetylase and as such a putative repressor of gene expression, we focused our subsequent analyses on the set of genes that was upregulated in the *hd2b* mutant. Comparative analysis between the *hd2b* and *mpk3* transcriptomes revealed an overlap among the identity of the deregulated genes in the two mutants. Indeed, a total of 414 genes were commonly upregulated in the two mutants (Fig. 2b) representing 32% of the *mpk3* upregulated genes. This result was not surprising because MPK3 likely has many substrates in addition to HD2B that contribute to the gene expression modifications observed in the transcriptome of our mutants. Reciprocally, HD2B likely is involved in diverse cellular processes that include, but are not limited to, pathogen defense. Gene ontology (GO) analysis of this class of genes revealed a significant enrichment in RNA biosynthetic processes and transcription (Fig. 2c).

To put these responses in the context of defense responses, the transcriptome results of untreated *hd2b* and *mpk3* mutants were compared with data obtained on WT plants after flagellin treatment, which allowed us to distinguish 150 genes that were upregulated in the *mpk3* and *hd2b* mutants and upon flagellin treatment (Fig. 2d), suggesting that the HD2B-MPK3 module normally represses the expression of these genes but that the inhibitory effect is alleviated during pathogen defense.

To further dissect the mechanisms by which MPK3 and HD2B regulate these genes, and based on the changes in their expression levels from the RNA-seq data, we selected four genes, *AT2G31290*, *AT5G03350*, *AT3G11380*, and *AT2G28180*, for further analysis. Quantitative reverse transcription polymerase chain reaction (RT-qPCR) confirmed the RNA-seq results indicating an over-expression of *AT2G31290*, *AT5G03350*, *AT3G11380*, and *AT2G28180* in *mpk3* and *hd2b* mutants (Fig. 2e).

Because HD2B is a histone deacetylase, we hypothesized that HD2B could be directly interacting with the gene loci to repress their transcription. To test if HD2B directly binds the four regulated genes, we performed chromatin immunoprecipitation (ChIP)-qPCR on seedlings expressing a GFP-HD2B fusion protein under the control of the endogenous HD2B promoter (*pHD2B::GFP-HD2B*). As a negative control, we included the *AT1G07690* gene that is upregulated in *hd2b* mutants (Additional file 1: Figure S13A) but was not identified as a HD2B target by ChIP-seq. A marked enrichment of HD2B binding on each of the four candidate genes but not on *AT1G07690* was found in the absence of flagellin treatment (Fig. 2f and Additional file 1: Figure S13B). Therefore, the HD2B protein is associated with chromatin and binds to these four genes, suggesting that HD2B directly regulates their transcription.

Acetylation and deacetylation are dynamic processes that can be rapidly reprogrammed, depending on the signals received by the cell. To determine the level of H3K9 acetylation, we performed ChIP-qPCR assays on these representative genes. Interestingly, we found hyper-acetylation on these four genes in both *mpk3* and *hd2b* mutants when compared with WT (Fig. 2g). Altogether, our results indicate that the HD2B-MPK3 module is required to regulate the basal expression of a subset of genes. In *hd2b* and *mpk3* mutants, the absence of deacetylation results in the constitutive induction of these genes (Fig. 2e). Thus, in addition to responding to pathogen stress, MPK3 activity appears to be required under normal conditions to promote HD2B-directed histone deacetylation to repress unwanted expression of these genes (Figs. 2e–g).

### HD2B is involved in pathogen defense

**Fig. 3:**
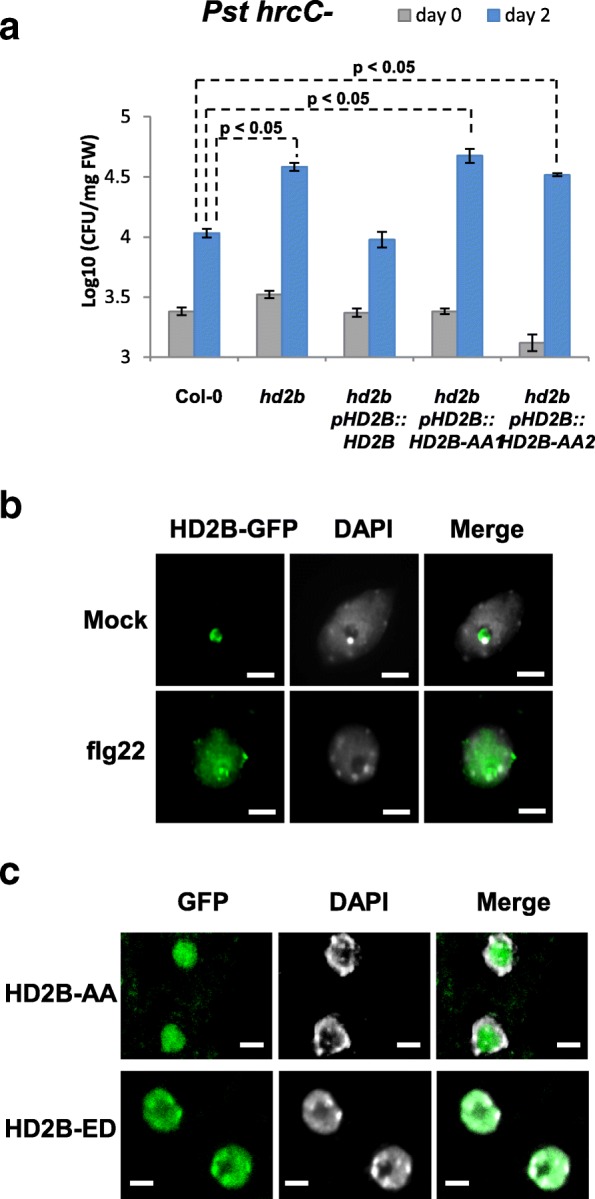
HD2B plays a role in plant immunity and shuttles from the nucleolus to the nucleoplasm. **a** Susceptibility of *hd2b* mutant and independent *hd2b* transgenic lines expressing an unphosphorylatable version of HD2B-AA (*hd2b pHD2B::GFP-HD2B-AA #1 and #2*) to *Pseudomonas syringae* pv. tomato hrcC- was compared to that of WT Col-0 and to a complemented mutant line (*hd2b pHD2B::GFP-HD2B*) as a control. Fourteen-day-old in vitro seedlings were incubated with a suspension of Pst hrcC-, and bacteria were quantified 2 h (day 0) and two days (day 2) after inoculation. *hd2b* mutant plants and *GFP-HD2B-AA* lines showed enhanced susceptibility compared with col-0 and with the non-mutated *GFP-HD2B* complemented line. Average values and standard deviations were calculated from three independent experiments. For each condition (day 0, day 2), a one-way ANOVA followed by an all pairwise multiple comparison procedure was performed (Holm-Sidak method, 23 > n > 8, *p* < 0.05). **b** Immunofluorescence staining was performed on *pHD2B::GFP-HD2B* rosette leaves either mock-treated or elicited with flg22 for 30 min. In mock-treated tissues, GFP-HD2B was localized to the nucleolus while GFP-HD2B was observed in the whole nucleus after flg22 treatment. Scale bars, 5 μm. **c** GFP-HD2B-ED that mimics a constitutively phosphorylated version of HD2B was stably expressed in Arabidopsis plants and accumulated in the nucleoplasm while GFP-HD2B-AA was localized in the nucleolus. Scale bars, 5 μm

Because the MPK3-HD2B module regulates pathogen response genes, we investigated the direct relevance of HD2B during biotic stress. In Arabidopsis, MPK3 has been shown to negatively regulate the basal activity of defense genes in the absence of MAMPs, but to be required for full expression of defense-related genes upon pathogen challenge [16]. Because MPK3 interacts and phosphorylates HD2B, we hypothesized that HD2B might also be implicated in defense against pathogens. To test this hypothesis, *hd2b* mutants were challenged with the non-pathogenic bacterial strain *Pseudomonas syringae* pv. tomato hrcC- that carries a mutation in the type 3 secretion apparatus and is hence deficient in effector deployment. Mutants showed increased sensitivity compared with WT plants (Fig. 3a). As a control, we also tested the *hd2b* mutant complemented by expression of *HD2B* under its own promoter (*pHD2B::GFP-HD2B*). *hd2b* complemented with *pHD2B::GFP-HD2B* showed infection levels of *Pst hrcC-* that were similar to WT col-0 plants. We also compared the resistance of the *hd2b* mutant and HD2B over-expressing lines with WT Col-0 plants upon infection to virulent *Pseudomonas syringae* pv. tomato *PstDC3000. hd2b* mutants were also more susceptible than WT, whereas plants over-expressing *HD2B* under the constitutive 35S cauliflower mosaic virus promoter (*35S::HD2B*) were more resistant to *Pst* DC3000 (Additional file 1: Figure S14). These results show that HD2B is implicated in the defense against pathogens probably by regulating defense genes. We hypothesized that HD2B is not involved in MAMP signaling upstream of the MAP kinase pathway. To confirm this, we first analyzed the MPKs activation after flg22 treatment in both WT and *hd2b* mutant and no changes were observed (Additional file 1: Figure S15). This result suggested that the signaling cascade is not affected in the *hd2b* mutant. We next asked if flagellin sensitivity is affected in this mutant. We did not observe any difference in growth inhibition (Additional file 1: Figure S16), suggesting that HD2B is not involved in MAMP-induced growth inhibition which is MAPK-independent.

### The HD2B phosphorylation status determines its sub-nuclear localization and choice of target genes

Having established that HD2B contributes to pathogen defense and that it can be phosphorylated by MPK3, we investigated in more detail the consequences of MPK3-dependent HD2B phosphorylation in response to flagellin. MPK3-mediated phosphorylation could affect HD2B activity in several ways, such as modulating its catalytic activity, its stability, or cellular localization. To discern if MPK3-mediated phosphorylation affects any of these parameters, we first investigated the subcellular localization of HD2B after flagellin-induced MPK3 activation. In p*HD2B::GFP-HD2B* leaves, GFP-HD2B was observed to be relocated from the nucleolus to the nucleoplasm (diffuse and speckle pattern) uniquely after flg22 recognition but not after mock treatment (Fig. 3b). This relocalization was confirmed also to occur in protoplasts (Additional file 1: Figure S17), indicating that HD2B moves from the nucleolus to other sites in the nuclear compartment in response to flg22 treatment. To examine the role of HD2B phosphorylation by MPK3 on the sub-nuclear localization of HD2B, we analyzed the localization of WT and mutated HD2B. Consistent with phosphorylation playing a role in HD2B localization, YFP-HD2B-ED (HD2B-T249E/S266D), a mutated form of HD2B that mimics a constitutively phosphorylated version of the HD2B protein, accumulated in the whole nucleus of Arabidopsis protoplasts even in the absence of flagellin treatment (Additional file 1: Figure S18). We confirmed this nuclear relocalization in stably transformed Arabidopsis lines expressing *pHD2B::GFP-HD2B-ED* (Fig. 3c and Additional file 1: Figure S19) suggesting that flg22-triggered MPK3 phosphorylation of HD2B induces the nuclear relocalization of HD2B. To test the role of HD2B phosphorylation in vivo, we introduced a non-phosphorylatable HD2B version HD2B-AA (HD2B-T249A/S266A) in the *hd2b* mutant. This mutated version of the protein accumulated only in the nucleolus (Fig. 3c). We next tested the sensitivity of HD2B-AA complemented *hd2b* lines to *P. syringae hrcC-*. These plants displayed enhanced sensitivity to the pathogen when compared to plants expressing WT *HD2B* (Fig. 3a), further confirming that phosphorylation of HD2B is required for proper activation of defense mechanisms. Consistently, the HD2B-AA protein fails to restore a normal expression level to HD2B target genes that are constitutively activated in the *hd2b* mutant (Additional file 1: Figure S20).

**Fig. 4:**
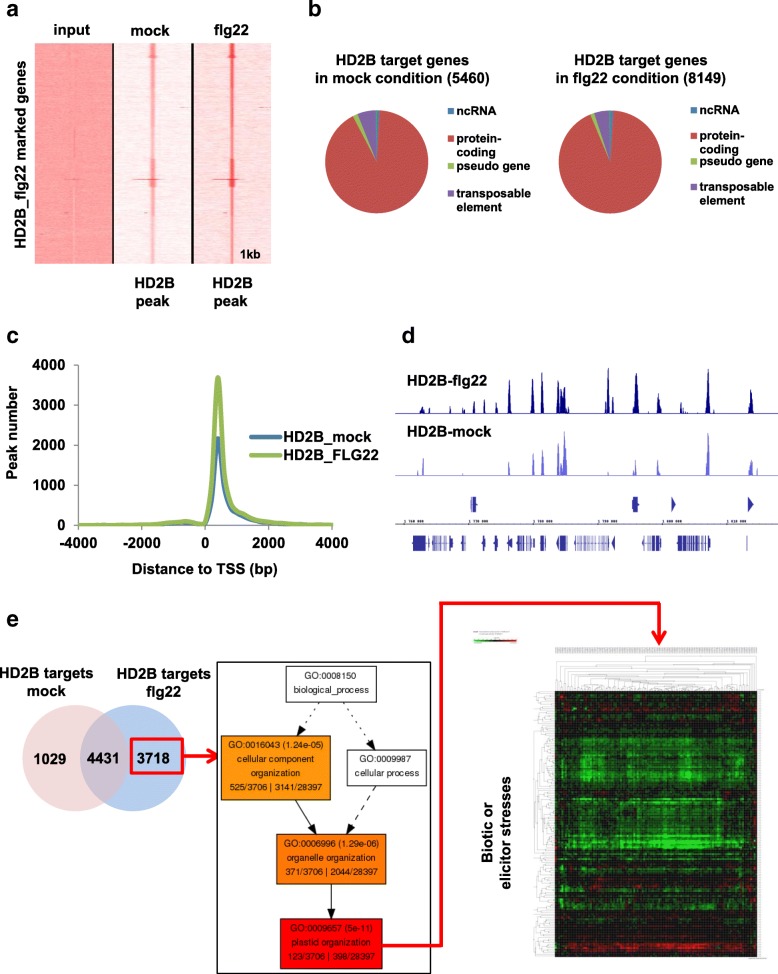
Flagellin recognition leads to HD2B redistribution to a new set of genes. **a** Comparison of HD2B tag density in the regions of ± 1 kb around the HD2B-occupied loci upon flg22 treatment. HD2B binding was determined by ChIP-seq experiments with an anti-GFP antibody in *pHD2B::GFP-HD2B* seedlings upon either mock or flg22 treatment. A comparison with the Input tag density confirmed HD2B enrichment after ChIP-seq in mock and flg22 conditions. **b** *Pie chart* representation of the classification of HD2B target genes upon mock or flg22 treatment. Genomic annotation of the detected HD2B peaks was performed using the Genomic Position Annotation Tool (GPAT). **c** HD2B global profiles. The majority of HD2B peaks were located at approximately 300 bp downstream of the transcription start site. HD2B peaks in both mock-treated and flg22-treated plants are represented to compare their relative position to the TSS. **d** Genome Browser view of ChIP-seq data across a region of chromosome 3 targeted by HD2B. **e** Flagellin treatment induces HD2B targeting to a new set of genes involved in plastid organization which is repressed by biotic stresses. The *Venn diagram* represents the overlap between HD2B targets in mock and flg22 conditions (*left*). The hierarchical tree graph shows a significant enrichment of the specific flg22-HD2B-targets in “plastid organization” with Agrigo GO Analysis Toolkit (*middle*). The *heatmap* generated with publicly available microarray data and the Genevestigator software 56 indicates that specific flg22-HD2B target genes are repressed after biotic or elicitor stresses. Each *column* represents a gene from the list and each *line* represents a particular microarray experiment. *Red*, *green*, and *black* colors indicate upregulation or downregulation or no change of gene expression, respectively (*right*)

To determine if the change in HD2B localization has an impact on the genes that this HDAC targets, we performed ChIP-seq in *pHD2B::GFP-HD2B* seedlings after a 30-min flagellin or mock treatment. First, we controlled the HD2B enrichment after ChIP-seq (Fig. 4a) and then performed a peak detection using MACS2. In control mock conditions, HD2B bound 5460 genomic loci, whereas it bound 8149 loci in flg22-treated plants (Additional file 2: Table SIV), indicating that flg22 treatment induced a recruitment of HD2B on chromatin. These differences could be attributed to a relocalization of the protein rather than to modifications of its accumulation since the expression of *HD2B* remains constant upon flagellin treatment (Additional file 1: Figure S21). 4431 HD2B target sites overlapped between mock- and flg22-treated plants, revealing a redistribution of HD2B on chromatin.

In mock-treated and flg22-treated conditions, most HD2B peaks were found approximately 300 base pairs (bp) downstream of the transcription start site (TSS) (Fig. 4c and d), and most target genes (84% in mock-treated and 78% in flg22-treated plants) were protein-coding genes (Fig. 4b). Several of these ChIP-seq identified targets were confirmed by ChIP-qPCR in this study (Fig. 2f). A GO analysis of the protein-coding HD2B-targeted genes revealed a significant enrichment for genes involved in defense response under mock conditions, whereas HD2B targeted genes after flg22 treatment were mainly involved in plastid organization and interestingly these genes are downregulated after a pathogen attack (Fig. 4e and Additional file 1: Figure S22).

To correlate HD2B binding to changes in gene expression, we compared the ChIP-seq results obtained for HD2B binding sites with the transcriptome of the *hd2b* mutant. In total, 277 of HD2B direct targets were upregulated in the *hd2b* mutant, whereas only 79 were downregulated (Additional file 1: Figure S23). This low level of correlation could be explained by the redundancy in the HD2 protein family. Together, these results indicate that after pathogen recognition, MPK3 modulates HD2B localization and activity to promote its dynamic redistribution on a new set of target genes.

### Modulation of H3K9ac levels is a hallmark of the flagellin response

Because HD2B targets are a highly varied set of genes before and after flg22 treatment, it is possible to assume that changes in histone acetylation are an important component of the cellular response to pathogens. To analyze the contribution of histone acetylation to the flg22-induced immune response, we analyzed the H3K9ac landscape before and after 30 min of flg22 treatment using a ChIP-seq approach. We identified about 15,000 peaks both in control and flg22-treated plants (Additional file 3: Table SIII). Most of these peaks were observed at core promoters or protein-coding genes where they predominantly localized to the first nucleosome after the TSS (Additional file 1: Figure S24A–E). According to the agriGO toolkit, genes harboring the H3K9ac mark were associated with developmental processes, responses to abiotic and biotic stimulus, signaling cascades and responses to hormone stimuli, both in control conditions and after flg22 treatment (Additional file 1: Figure S24F).

**Fig. 5:**
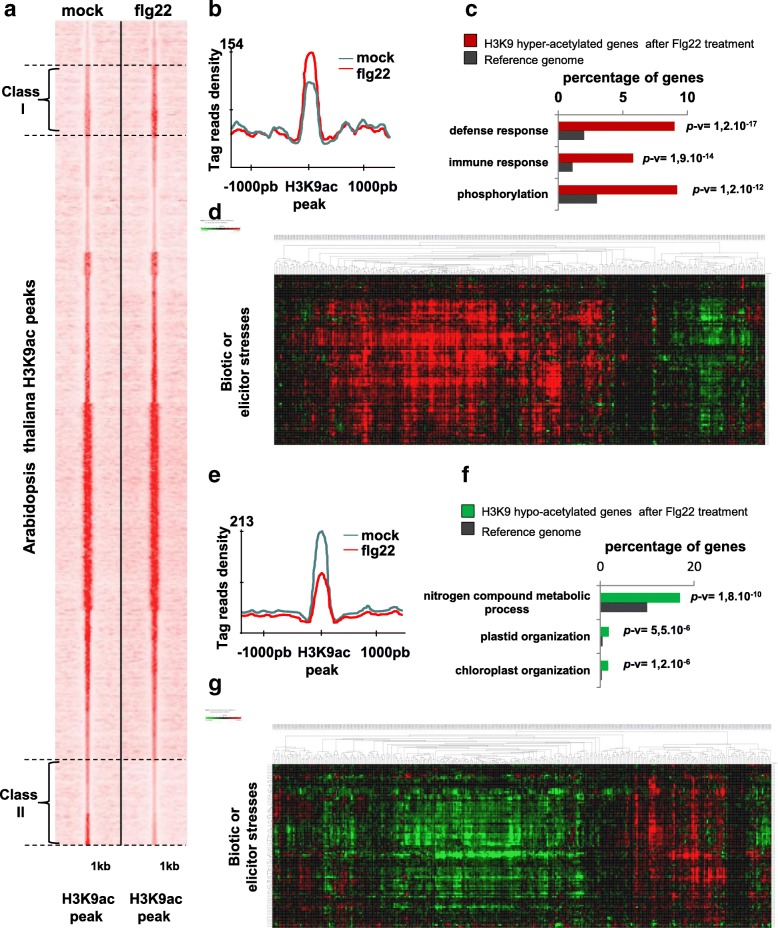
Flagellin recognition modulates the genome-wide H3K9 acetylation landscape. **a** Flagellin triggers hyper- and hypo-acetylation of two different peak clusters. H3K9ac ChIP-seq data from mock- and flg22-treated plants were compared using seqMINER. From these analyses, three classes of peaks emerged: classes I and II contain hyper- and hypo-acetylated peaks, respectively, after flg22 treatment, and the third class contains peaks that do not change. **b** Tag read densities of class I H3K9ac peaks are stronger upon flg22 treatment. The *graph* represents the average of tag reads relative to the H3K9 peak summit positions for all the peaks from cluster I. **c** GO analysis of H3K9 flg22 hyper-acetylated genes. Genomic annotation of the H3K9 hyper-acetylated peaks was performed using the GPAT. GO data were extracted with the Agrigo GO Analysis Toolkit. A *histogram* of the values highlights the enrichment of the GO classes. *P* values for each enriched class are indicated (*p*v). **d** H3K9 flg22 hyper-acetylated genes mainly are induced by biotic or elicitor stresses. The gene expression of flg22-induced H3K9 hyper-acetylated genes was analyzed using publicly available microarrays data and the Genevestigator software [60]. Each *column* represents a gene from the list and each *line* represents a particular microarray experiment. *Red*, *green*, and *black* colors indicate an upregulation, downregulation, or no change of gene expression, respectively. **e** Tag read densities of class II peaks are stronger in mock conditions. The *graphic* represents the average of tag reads relative to the H3K9 peak summit positions for all the peaks from cluster II. **f** GO analysis of H3K9 flg22 hypo-acetylated genes. Genomic annotation of the H3K9 hypo-acetylated peaks was performed as in Fig. 5c. A *histogram* highlights the enrichment of the GO classes. *P* values for each enriched class are indicated (*p*-v). **g** H3K9 flg22 hypo-acetylated genes are repressed mainly by biotic or elicitor stresses. The gene expression of flg22-induced H3K9 hypo-acetylated genes was analyzed and displayed as in (**d**)

We next compared the two ChIP-seq datasets using seqMINER and diffReps, which allows qualitative and quantitative comparisons to be made between a reference set of genomic positions and multiple ChIP-seq datasets [17]. Using this approach, we observed three classes of genes. The first class, consisting of 731 genes (Additional file 3: Table SIII), was hyper-acetylated after flg22 treatment (Fig. 5a and b). GO analysis of this class revealed a significant enrichment in genes involved in defense and immune responses and phosphorylation (class I, Fig. 5c and Additional file 1: Figure S26A). The second class of 13,159 genes did not display any obvious changes in H3K9ac marks (class II, Fig. 5a). The third class totaling 787 genes was hypo-acetylated after flg22 treatment (class III, Fig. 5a and e and Additional file 3: Table SIII) and mainly consisted of genes involved in chloroplast and plastid organization and in metabolic processes (Fig. 5f and Additional file 1: Figure S26B). Gene expression analysis revealed that most hyper-acetylated genes were induced (Fig. 5d) and most hypo-acetylated genes were repressed by diverse biotic stresses (Fig. 5g), providing strong evidence for a positive correlation between the acetylation and the expression level of biotic stress responsive genes. Consistently, comparing these results with the transcriptome of WT plants treated with flg22 revealed that about 98% of hyper-acetylated genes were induced by flg22 treatment, whereas only 2% were repressed, and about 64% of hypo-acetylated genes were repressed by flg22 treatment, whereas only 36% were induced (Additional file 1: Figure S27), clearly showing that H3K9ac is associated with gene activation. Our results indicated that flg22-induced genes were regulated mainly by de novo acetylation, whereas flg22-repressed genes were regulated by dynamic deacetylation, highlighting the role of HDAC proteins in the control of flg22-regulated genes.

### HD2B is a major contributor of flg22-induced changes in histone acetylation

**Fig. 6:**
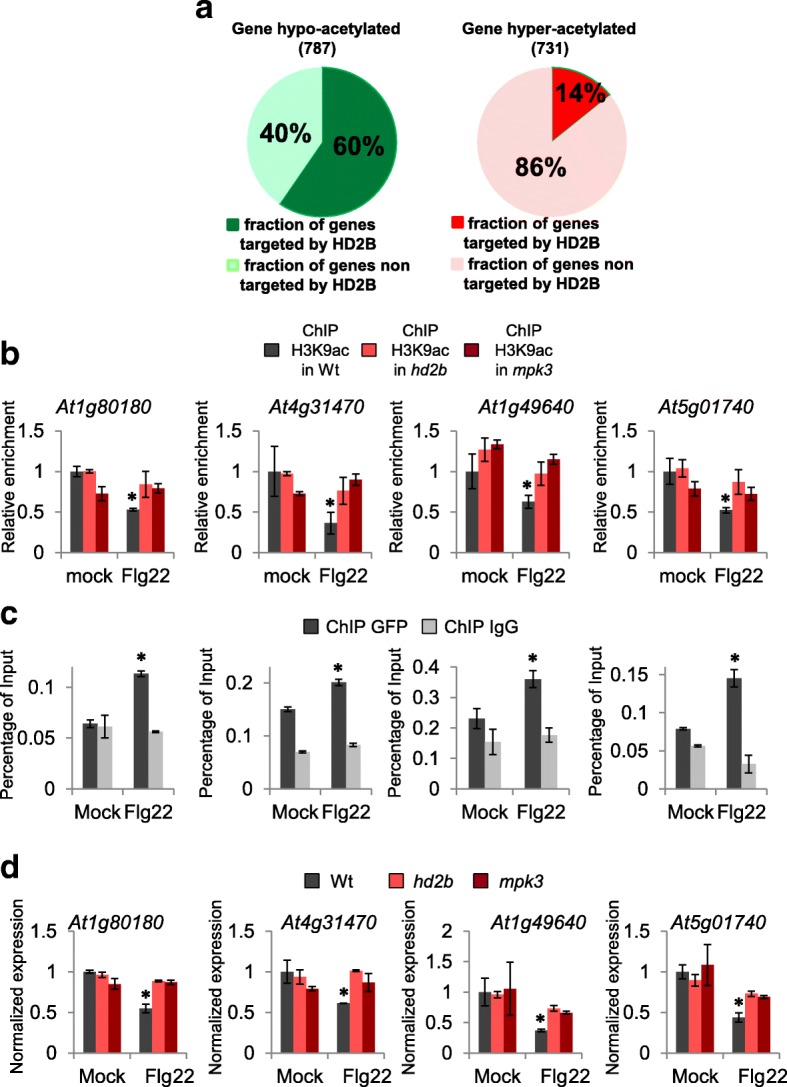
The MPK3-HD2B module contributes to the regulation of H3K9 acetylation dynamics in response to flagellin. **a** Sixty percent of H3K9 flg22-induced hypo-acetylated genes are HD2B targets (*left*) compared with only 14% of H3K9 flg22-induced hyper-acetylated (*right*). **b** MPK3 and HD2B promote H3K9 deacetylation of hypo-acetylated genes after flg22 treatment. ChIP-qPCR assays on four selected loci using an anti-H3K9ac antibody were performed in WT and *hd2b* and *mpk3* mutant seedlings either mock- or flg22-treated for 30 min. The four genes were hypo-acetylated after flagellin treatment in WT plants and this deacetylation failed to occur in the *hd2b* or *mpk3* mutants. *Asterisks* indicate significantly different values (ANOVA test, *p* < 0.05). **c** HD2B is bound to hypo-acetylated genes after flg22 treatment. ChIP-qPCR assays with anti-GFP antibodies were performed on *pHD2B::GFP-HD2B* seedlings using oligonucleotides in the proximal promoter region of the four selected loci. IgG antibody was used as a negative control. *Asterisks* indicate significantly different values (Student’s t-test, *p* < 0.05). **d** MPK3 and HD2B are required for gene repression of hypo-acetylated genes after flg22 treatment. Expression of the four selected genes was analyzed by qRT-PCR in WT, *hd2b*, and *mpk3* seedlings mock- or flg22-treated for 30 min. Genes were repressed in the WT but not in *hd2b* and *mpk3* mutants. *Asterisks* indicate significantly different values (ANOVA test, *p* < 0.05)

Because histone acetylation appears to be a major factor controlling the transcriptional reprogramming induced by pathogen recognition, we investigated whether HD2B could be a central contributor to this process. Among the 787 genes hypo-acetylated after flg22 treatment, 60% were HD2B targets, indicating that HD2B plays an important role in the transcriptional reprogramming induced by pathogens (Fig. 6a). As expected, among the 731 hyper-acetylated genes only 14% were HD2B targets (Fig. 6a).

To clarify the mechanism by which the MPK3-HD2B module acts on genes after flg22 treatment, we selected four HD2B target genes that were hypo-acetylated after flg22 treatment. We first confirmed their acetylation status (Fig. 6b) and then verified that they were bound by HD2B after flg22 treatment (Fig. 6c). Second, we analyzed their expression and acetylation levels before and after flg22 treatment in WT plants and compared them with the levels in *mpk3* and *hd2b* mutants (Fig. 6d). As expected, in WT control plants, hypo-acetylated genes were repressed and were further hypo-acetylated after flg22 treatment, but neither gene repression nor a change in acetylation occurred in the *mpk3* and *hd2b* mutants, confirming the role of the MPK3-HD2B module in the repression and acetylation dynamics of these genes (Fig. 6b–d). Furthermore, to functionally prove that HD2B phosphorylation plays a major role in this process, we analyzed the expression levels of these specific four genes by qRT-PCR in our HD2B-AA line. We observed that this repression is not restored in the HD2B-AA line, providing further evidence for the functional relevance of HD2B phosphorylation (Additional file 1: Figure S28).

## Discussion

Plants are sessile organisms that constantly have to respond to changes in environmental conditions such as biotic or abiotic stress. In the case of biotic stresses, plants have adapted the capacity to recognize pathogens through MAMPs via specific receptors. The multi-faceted responses of plants to pathogens have been examined extensively and these studies have revealed that receptor-mediated pathogen recognition triggers MAP kinase signaling cascades, which, through the activation and repression of large gene sets, ultimately result in the establishment of MAMP-triggered immunity.

Previous studies have identified several transcription factors targeted by MAP kinases. For example, the bZIP (basic leucine zipper) transcription factor VIP1 (VirE1-INTERACTING PROTEIN 1) is specifically phosphorylated by MPK3 upon flg22 treatment and activates a number of defense-related genes such as *PR1* (*PATHOGENESIS*-*RELATED 1*) [10, 18]. However, extensive changes in the transcriptional program of cells likely rely not only on the activation of specific transcription factors but also on chromatin modifications that can dynamically and reversibly regulate gene expression. Consistently, we show here that flagellin-activated MAP kinases interact with and phosphorylate the histone deacetylase HD2B to control the expression level of biotic stress-regulated genes through modulation of the H3K9ac histone mark.

To date, a handful of histone modifiers have been implicated in plant innate immunity. For example, salicylic acid (SA) signaling plays an essential role in plant pathogen resistance and is controlled partially by the HDAC SIRTUIN2 (SRT2), which represses the expression of several SA biosynthetic genes such as PAD4 and SID2 [19]. Consistently, *srt2* mutant Arabidopsis plants were reported to be more resistant to pathogen infection than WT control plants, whereas an SRT2 over-expressing line was more susceptible. In addition, it was reported in Arabidopsis that mutations in the HDAC *HDA19* result in enhanced basal expression of several biotic responsive genes [20] and improve tolerance to *P. syringae* [21], although contradictory results have been described previously [22]. Moreover, the rice HDAC HDT701 negatively regulates innate immunity by directly binding and modulating the histone H4 acetylation levels of PRR and defense-related genes [23].

Here, we measured the resistance of *hd2b* mutants to the both virulent and non-pathogenic *Pseudomonas syringae* strains and concluded that *hd2b* mutants were more susceptible than WT control plants, whereas *HD2B* over-expressing plants were more resistant. Hence, different HDACs can lead to similar or contrasting outcomes for plant immunity likely depending on the genes they target; our results and those reported by others clearly highlight the crucial contribution of chromatin regulation to pathogen defense.

Although several studies provide evidence for the involvement of histone modifiers in pathogen response, the way in which their activity is connected to biotic stress signaling cascades has not been unraveled. In this work, we connected MAPK-induced signal transduction to HDAC-dependent histone modifications. Indeed, our results show that H2DB is a direct target of MAPKs and that HD2B regulates a large number of genes involved in pathogen defense. Although our study is the first to describe a general histone modifying protein as a direct target of a MAPK in plants, such mechanisms seem to be highly conserved in eukaryotes. Indeed, in animals, MAPKs drive histone modifications and direct chromatin remodeling. For example, phosphorylation of the histone acetyl-transferase p300 by the MAP kinase ERK2 promotes its localized histone binding [24]. Likewise, ERK1 and ERK2 modulate the assembly of chromatin remodeling complexes and, thereby, control the expression of vitamin D-responsive genes [25].

The Arabidopsis genome codes for 18 HDACs, which fall into four groups [26, 27]: class I and II HDACs correspond to yeast RPD3 and HDA1, respectively, class III enzymes are NAD-dependent HDACs related to yeast SIR2, and class IV HDACs are plant-specific and consist of four members called HD2A-D [26]. The four class IV HDACs all contain a conserved N-terminal catalytic and a central acidic domain and a divergent C-terminal region that suggest specific functioning of the different proteins [26]. In contrast to the other three Arabidopsis HD2s, HD2B does not contain a Zn-finger motif in its C-terminus. However, with the exception of Arabidopsis HD2A, the two MAPK-targeted C-terminally located HD2B in vivo phosphorylation sites are highly conserved in other species, including the equivalent rice and maize HD2s [26]. Our data indicate that phosphorylation of these sites is important for the molecular function of HD2B and that the phosphorylation status of the two residues determines the intra-nuclear localization of the HD2B. Possibly, a nucleolar protein could anchor unphosphorylated HD2B to this sub-nuclear compartment and MAPK-directed phosphorylation of HD2B could release HD2B to enter the remaining nuclear compartment and associate with novel chromosomal loci. In this way, the nucleolus could serve as a stocking center to prevent HD2B from potentially associating with certain labile nuclear sites and could provide a protein synthesis-independent mechanism to make HD2B rapidly available for gene regulation upon signaling.

Our results show that MPK3 likely modulates HD2B activity by altering its nuclear distribution. Indeed, flagellin treatment induces the relocalization of HD2B in an MPK3-dependent manner, and this effect can be obtained by expressing a phosphomimetic version of HD2B. Consistently, control of the intracellular location has been described in yeast and mammals as an important regulatory mechanism of HDAC activity [28, 29]. As expected from their ability to deacetylate histones, HDACs are found generally in the nucleus of most organisms. Nevertheless, in mammals, the Class IIa HDACs were found both in the cytoplasm and in the nucleus. Indeed, specific phosphorylation of HDAC7 induces its nuclear exclusion, rendering it unable to impact transcription [30]. In the case of HD2B, phosphorylation leads to a sub-nuclear relocalization from the nucleolus to the nucleoplasm in specific chromatin regions. In agreement with the flg22-induced relocalization of HD2B, our ChIP-seq analyses revealed a drastic shift in the HD2B chromatin targeted sites upon flagellin treatment. In mammals, it has been reported that the phosphorylation of HDAC2 leads to its redistribution on chromatin and increased recruitment to promoters [31, 32], suggesting that HDAC phosphorylation could be a mechanism conserved from mammals to plants to drive their redistribution in response to a stimulus.

HD2B is a HDAC and is predicted to function as a repressor of gene expression. As expected, a proportion of HD2B target genes were constitutively upregulated in *hd2b* mutants (Additional file 4). Furthermore, 64% of the genes that are hypo-acetylated after flagellin treatment are direct HD2B targets, indicating that HD2B contributes substantially to MAMP-triggered transcriptional reprogramming (Additional file 5). The study of pathogen-induced histone modifications is a fledgling field of research. In mammals, although four bacteria have been reported to modulate histone acetylation levels upon infection, the underlying mechanisms are not known [33–36]. In plants, modulation of histone acetylation levels in response to biotic stress has also been described, but again the underlying mechanisms remained unclear [19, 21, 23, 37, 38]. Our data show that genome-wide modulation of H3K9ac levels is an early chromatin response to a biotic stress. Histone acetylation is a labile chromatin mark [39, 40] and, as such, is a fast and reversible post-translational process that can allow plants to rapidly modulate gene expression. Thus, this mark is ideally suited for biological programs that require an immediate response to fluctuating environmental conditions. In line with this characteristic, we observed a strong correlation between upregulation of gene expression and hyper-acetylation, whereas downregulation correlated with hypo-acetylation. Altogether, our data suggest that modulation of the genome-wide H3K9ac landscape is a hallmark of the flg22 response and that HD2B is a key player in this process. However, our studies do not exclude the involvement of additional HDACs in this process.

Finally, our detailed analysis of the expression levels, HD2B binding, and histone acetylation of HD2B target genes under control and flg22 treatment conditions revealed that HD2B has several roles. We have shown that HD2B regulates the basal expression level of a subset of genes in the absence of pathogen challenge, probably by acting as a counter-balance to the activity of HATs. Indeed, expression of these HD2B-targeted genes in unchallenged plants is constitutively high in *hd2b* mutants. These genes can be distributed into two classes after flagellin treatment: class I genes are repressed by flagellin due to increased HD2B recruitment on these sites, whereas class II genes are induced by flagellin due to eviction of HD2B from these loci.

In summary, our work defines the first example of a MAP kinase-regulated chromatin mechanism and details how MAMP-triggered MAP kinase signaling regulates global changes in the chromatin landscape. As such, it sets the stage for other large-scale studies examining the contribution of protein kinase-mediated chromatin regulation in plants and mammals.

## Methods

### Plant material, growth conditions, and treatments

T-DNA insertion lines *hd2b* (At5g22650) from SAIL collection [41] Sail_1247_A02 (Additional file 1: Figure S29) and *mpk3* SALK_151594 [42] were obtained from the Nottingham Arabidopsis Stock Centre (NASC).To produce transgenic plants expressing a 35S::GFP:MPK3 construct, the coding region of MPK3 (At3g45640) was amplified from cDNA of Col-0 by PCR using the following primers: forward, 5′gc gga tcc atg aac acc ggc ggt ggc3′, reverse 5′gc act agt cta acc gta tgt tgg ctt gag3′. The restriction enzyme sites, set in bold, that had been added to the primers were utilized to ligate the open reading frame to BamHI/SpeI sites of pCAT-GFP, resulting in *p35S::GFP-MPK3*. For the generation of stably transformed *A. thaliana* plants, the expression cassette was excised by Sse8387I and cloned into the PstI site of the binary vector pCB302 [43]. This vector was transformed to *A. tumefaciens* strain GV3101 by electroporation. *A. thaliana* Col-0 plants were transformed by floral dip method according to Clough and Bent [44]. Transformed plants were selected on BASTA.

Both *pHD2B::GFP-HD2B* and *35S::GFP-HD2B* constructs were made by using Gateway technology (Invitrogen). The CDS of HD2B was PCR amplified from cDNA with forward primer 5′-caccATGGAGTTCTGGGGAGTTGC-3′ and reverse primer 5′-AGCTCTACCCTTTCCCTTGC-3′ using Phusion High Fidelity DNA polymerase (New England BioLabs). The putative promoter (1.055 kb upstream of the start codon) was PCR amplified from genomic DNA with forward primer 5′-caccGTTTTGGATCTGCAGACAAGG-3′ and reverse primer 5′-TGTTGTTGAACGAGGAAGAGAG-3′. Both PCR fragments were cloned into pENTR/D-TOPO (Invitrogen). The promoter was then recloned from the pENTR/D-TOPO into a pENTR4-1 vector (Invitrogen) in front of a GFP using the NotI and AscI restriction sites. The pENTR4-1 containing the HD2B promoter and GFP, the pENTR-D-TOPO-AtHD2B vector and a pENTR2-3 vector containing a CaMV 35S terminator were recombined by a multisite gateway reaction (Invitrogen) into the binary destination pBnRGW vector. This is a modified vector based on pKGW [45] in which the kanamycin resistance was replaced with basta resistance and the NAP::DsRed expression cassette from pFluar 101 [46] was introduced for easy selection of red fluorescent transformed seeds. To create *35S::GFP-HD2B* construct the same strategy was used but pENTR4-1 contained CaMV35S promoter. *A. tumefaciens* strain C58 mediated transformation for both constructs was performed as described by Bechtold and Pelletier [47]. Seed-specific expression of red fluorescent protein DsRed permitted the identification of mature transformed seeds by fluorescence stereo microscopy (Leica).

For flagellin experiments, seeds were surface-sterilized by treatment with bayrochlore and then soiled in sterile half-strength MS liquid medium, placed for 2–4 days at 4 °C to obtain homogeneous germination, and plants were grown in chambers at 20 °C in long-day (16 h of light) conditions. After 14 days, flg22 peptide was added in the medium to a final concentration of 1 μm when necessary.

Infection assays in Arabidopsis rosette leaves with *Pseudomonas syringae* pv. tomato PstDC3000 were performed as described previously [48] on leaves of 4–5-week-old plants grown on soil in environmental chambers at 22 °C under short-day conditions (8 h of light).

For Pseudomonas pathogen assays in Arabidopsis seedlings, 0.5 MS plates containing 14-day-old Arabidopsis plantlets were flooded with a bacterial suspension of the hrcC- mutant (defective in type III secretion system) of *Pseudomonas psyringae* pv. tomato DC3000 for 3 min. The bacteria were grown ON at 30 °C in LB broth + Rif and resuspended in 10 mM MgCl_2_ after several washings for the removal of the media and the antibiotic_._ The final concentration of the suspension was adjusted to an OD_600_ = 0.1 and supplemented with 0.025% Silwet L-77. After discarding the bacterial suspension, the plates were sealed with micropore and put back in the incubation room. Day 0 samples were taken 2 h post-inoculation and day 2 samples after 48 h. For the sampling process, the rosettes of four plantlets were cut and weighed together, registering each time the fresh weight in milligrams. Afterwards, the rosette surface was sterilized by a 5-s wash in 70% ethanol, followed by two washes in sterile water. The samples were ground in order to release the bacteria and serial dilutions performed. A total of 10 μL of each dilution was plated on plates with LB + Rif and incubated for two days at 30 °C. The growth of the bacterial population inside the plantlets was determined by calculating the total amount of bacterial CFU per mg of fresh weight. Each experiment was performed in quintuplicate, analyzing eight samples per accession on each experiment.

### Kinase assays

Full-length *HD2B* (At5g22650) was amplified and cloned into pGEX4T-1. For mutagenesis of HD2B, PCR was performed on plasmids with Pfu Ultra. PCR mixtures were digested for 2 h with *Dpn*I and transformed into *Escherichia coli*. Clones were sequenced and each mutation was transformed into *E. coli* BL21. GST protein expression was induced for 4 h at 37 °C with 1 mM IPTG and purified according to the manufacturer’s protocol. For producing HD2B T249E and S266D, the following primers were used: GGAGGACACACCGCC**GA**ACCACACCCAGCT, AGCTGGGTGTGGT**TC**GGCGGTGTGTCCTCC, GTGAATGCTAACCAG**GA**CCCCAAGTCTGGA, and TCCAGACTTGGGG**TC**CTGGTTAGCATTCAC, respectively.

Radioactive kinase assays were performed with MPK3, 4, and 6 immunoprecipitated from extracts of *A. thaliana* cell cultures that were treated for 10 min with 1 μM flg22. Non-radioactive kinase assays for phosphosite mapping were performed the same for 45 min at 30 °C.

### Protein localization in protoplasts

Full-length HD2B and HD2B-DD mutated forms were cloned in a pGreen plasmid behind a YFP gene driven by a 35S promoter. Arabidopsis protoplasts were prepared from a suspension culture as described [49]. One day after transformation, flg22 peptide was added in the medium to a final concentration of 2 μM for 15 min when necessary and localization was checked by fluorescence microscopy.

### Immunofluorescence labeling

Leaves of plants stably transformed with *p35S::GFP-HD2B* construct were treated for 30 min with or without 1 μM flg22 and fixed in PFA 4% in PHEM (PIPES 60 mM; HEPES 25 mM; EGTA 10 mM; MgCl_2_ 2 mM pH 6.9) for 1 h at room temperature (apply vacuum 20 min to facilitate uptake of the fixation solution). Seedlings were washed 5 min in PHEM and 5 min in phosphate buffered solution (PBS) pH6.9 and chopped on a Petri dish in PBS supplemented with 0.1% Triton X-100 (w/v). The mixture was filtered (50 μm) and centrifuged for 10 min at 2000 g. The supernatant was carefully removed and the pellet washed once with PBS, gently resuspended in 20 μL PBS, and a drop was placed on a poly-lysine slide and air dried. Slides were rehydrated with PBS and permealized twice by 10 min incubation in PBST (PBS, 0.1% Tween-20 v/v). Slides were placed in a moist chamber and incubated overnight at 4 °C with primary antibody anti-GFP (Clontech, ref. 632592) in PBST supplemented with BSA (3% w/v). Slides were washed 5 × 10 min in PBST (at RT) and incubated for 1 h at room temperature in the dark with the secondary antibody (A11037 Invitrogen, Alexa Fluor 594 goat anti-rabbit) diluted (1/400 v/v) in PBST, 3% BSA. Slides were washed 5 × 10 min in PBST and then mounted with a drop of Vectashield with DAPI and observed as described for pollen mitosis analysis with the suitable cube fluorescence filters (BP340-380, DS 400, BP 450-490 for DAPI) (BP570-590, DS595, BP 605-655 for A594).

For whole mount immunofluorescence labeling, six-day-old p*HD2B::GFP-HD2B* seedlings were labeled according to the method of Sauer et al. [50] with small modifications: seedlings were fixed in 1.5% paraformaldehyde and 0.5% glutaraldehyde in 0.5 MTSB buffer (50 mM K-PIPES, 5 mM MgSO_4_-7H_2_0, 5 mM EGTA) at pH 6.8; cell-wall digestion enzyme mixture contained 1% meicelase, 1% cellulase, and 1% macerozyne in PBS; samples were incubated with rabbit polyclonal anti-AtMPK3 (SIGMA-ALDRICH) primary antibody diluted 1:350 in PBS supplemented with 2% BSA at 4 °C overnight, and subsequently with secondary antibody Alexa-Fluor 546 goat anti-rabbit IgGs (H + L) (Invitrogen) diluted 1:500 in PBS containing 2% BSA for 3 h (1.5 h at 37 °C and 1.5 h at room temperature). Finally, samples were counterstained with DAPI and mounted in one drop of 0.1% [w/v] para-phenylenediamine prepared in 90% [v/v] glycerol in PBS. Microscopic analysis of immunolabeled samples was performed using a Zeiss LSM710 (Carl Zeiss Jena, Germany). DAPI was excited at 405 nm and emission was detected between 410 and 476 nm. GFP was excited at 488 nm and emission was detected between 500 and 535 nm. Alexa 546-conjugated antibody was excited at 561 nm and fluorescence was detected between 566 and 591 nm. The post-processing of images was done using ZEN 2010 software, Photoshop 6.0/CS, and Microsoft PowerPoint.

### BiFC experiment

LR recombinations of *HD2B* and *MPK3* coding sequences in pENTR3C were done with split-YFP destination vectors pBiFC3 and pBiFC2 that allow N-terminal fusion with the C- and N-terminal YFP moieties, respectively [51]. Recombined vectors were transformed into the Agrobacterium C58C1 strain. *Nicotiana benthamiana* leaves were agro-infiltrated as previously described [52]. After three days, the YFP fluorescence was visualized using a confocal laser scanning microscope (Leica, Germany).

### Live-cell imaging

For microscopy, six-day-old plants stably transformed with *p35S::GFP-MPK3* construct were transferred to microchambers mounted between microscopic slides and coverslips with one Parafilm layer as a spacer. The chambers were filled with liquid half-strength MS medium. Microscopic analysis was performed using a Zeiss LSM 710 confocal laser scanning microscope (Carl Zeiss Jena, Germany). All images were acquired using a 40X objective lens (NA 1.42). GFP was excited at 488 nm and detected between 500 and 535 nm. Post-processing of images was done with the aid of Zeiss ZEN software (Ver.2010b) and Microsoft PowerPoint applications.

### Cell fractionation

Two grams of 16-day-old Col-0 seedlings were ground in a mortar with liquid nitrogen and were resuspended in buffer A containing 2.5% Ficoll type 400 (F-4375, Sigma-Aldrich), 5% Dextran (D1662, Sigma-Aldrich), 0.4 M sucrose, 25 mM Tris-HCl pH 7.5, 10 mM MgCl_2_, 5 mM DTT (D0632, Sigma-Aldrich), protease inhibitors (Complete cocktail, Roche), and phosphatase inhibitors (1 mM NaF, 0.5 mM Na_3_VO_4_, 15 mM B-glycerophosphate, 15 mM 4-nitrophenyl phosphate, Sigma-Aldrich chemicals). After a few minutes’ incubation on ice, the samples were filtrated through one layer of 62-μm nylon mesh by centrifugating for 3 min at 212 g at 4 °C. Triton X-100 was then added to a final concentration of 0.5% and the samples were gently mixed and incubated on ice for 15 min. An aliquot was kept at this step, hereafter referred to as Input. The samples were then centrifuged for 5 min at 1500 g at 4 °C and an aliquot of the supernatants was kept at this step, hereafter referred to as Cytoplasmic fraction. The pellets were gently resuspended in buffer B (buffer A and 0.1% Triton X-100) and centrifuged for 5 min at 1500 g at 4 °C. The pellets were then resuspended in buffer A and centrifuged for 5 min at 2000 g at 4 °C. The pellets were finally resuspended in SDS-sample buffer; they are hereafter referred to as nuclear fractions. SDS-sample buffer was also added to the Input and Cytoplasmic fractions and all three kinds of aliquots were heated at 95 °C for 10 min for complete denaturation. Protein samples were resolved by SDS-PAGE at a constant amperage of 15 mA per gel and transferred onto methanol-activated PVDF membranes (GE Healthcare) for 1 h at a constant voltage of 100 V. Blots were blocked with 5% non-fat dry milk in 1× TBST for 1 h and probed with primary antibodies overnight at 4 °C: anti-H3 (Abcam ref. Ab1791, diluted 1:10,000), anti-PEPC (Tebu-bio ref. 100-4163, diluted 1:15,000), and anti-MPK3 (described in [53], diluted 1:4000). The membranes were washed four times with 1× TBST. Goat anti-rabbit antibodies, at a dilution of 1:20,000 in 5% non-fat dry milk in 1× TBST, conjugated to horseradish peroxidase (A6154, Sigma-Aldrich), were used as secondary antibodies for 1 h at room temperature. The membranes were washed again four times with 1× TBST and the antigen-antibody interaction was detected with enhanced chemiluminescence reagent (ECL Prime, GE Healthcare) using a GeneGnome imaging system (Syngene).

### Co-immunoprecipitation

For co-immunoprecipitation assays in protoplasts, protein extracts were prepared from protoplasts as described [54]. For co-immunoprecipitation, samples were extracted in 50 mM Tris-HCl, pH 7.8, 150 mM NaCl, 1 mM EDTA, 0.1% Nonidet P-40 (NP-40), and proteinase inhibitor mix (Complete EDTA free, Roche). Three independent protoplast transformations of 100 μL each were pulled and used for protein extraction. Protein extracts were precleared with 15 mL of protein A–Sepharose beads for 2 h at room temperature, then immunoprecipitated overnight in the presence of antibodies with 25 μL of beads. Samples were washed three times with wash buffer (50 mM Tris-HCl pH 7.8, 150 mM NaCl, 5 mM EGTA, 5 mM EDTA, and 0.1% Tween 20) and subjected to immunoblotting.

For co-immunoprecipitation experiments *in planta*, transgenic lines expressing *p35S::GFP-HD2B* or *p35S::MPK3-c-Myc* constructs were crossed together to obtain plants expressing both constructs. Plant leaves were ground to a fine powder in liquid nitrogen. Cells were homogenized and lysed in Chris buffer containing 50 mM Tris-HCl pH 8.0, 0.5% NP-40, 200 mM NaCl, 0.1 mM EDTA, 10% glycerol, 10 mM NEM, and protease inhibitor mix (Complete EDTA-free, Roche). GFP-HD2B and MPK3-c-Myc were then immunoprecipitated using a polyclonal anti-GFP antibody (Clonetech) and a polyclonal anti-c-Myc antibody (Sigma), respectively. Immune complexes were collected by incubation for 2 h at 4 °C with Protein A/G Ultralink Resin (Thermo Scientific) and washed six times in lysis buffer. Immunoprecipitated proteins were detected by immuno-blot using a polyclonal anti-c-Myc antibody (Sigma).

### Immunoblotting

About 200 mg of 14-day-old seedlings were ground with liquid nitrogen and resuspended in 500 μL of a buffer containing 50 mM Tris-HCl pH 7.5, 150 mM NaCl, 0.1% NP40, 5 mM EGTA, 0.1 mM DTT (Sigma-Aldrich chemicals), protease inhibitors (Complete cocktail, Roche), and phosphatase inhibitors (1 mM NaF, 0.5 mM Na_3_VO_4_, 15 mM β-glycerophosphate, 15 mM 4-nitrophenyl phosphate, Sigma-Aldrich chemicals). The suspension was centrifuged at 20,000 g for 15 min at 4 °C, and the supernatant was collected. Protein quantification was carried out by Bradford method (Protein Assay Kit, Thermo Fisher Scientific) and the normalized protein amounts of all samples were denatured with SDS-sample buffer by boiling them at 95 °C for 10 min. Protein samples were resolved by SDS-PAGE and transferred onto PVDF membranes (Bio-Rad). Blots were blocked with 5% BSA in 1× TBST and then probed with Phospho-p44/42 MAPK (Erk1/2) (Thr202/Tyr204) (D13.14.4E) XP rabbit monoclonal antibody (#4370, Cell Signaling), hereafter referred to as anti-pTpY antibody. Goat anti-rabbit antibodies conjugated to horseradish peroxidase were used as secondary antibodies (A6154, Sigma-Aldrich). The antigen − antibody interaction was detected with chemiluminescent reagents (Clarity ECL substrate, Bio-Rad) using an imaging system (ChemiDoc MP System, Bio-Rad). Coomassie blue staining of blots was then carried out for protein visualization.

### Gene expression analysis

Total RNA was extracted from seedlings with the RNeasy MiniPrep kit (Qiagen), according to the manufacturer’s instructions. First strand cDNA was synthesized from 2 μg of total RNA using Improm-II reverse transcriptase (A3802, Promega) according to the manufacturer’s instructions. of the synthesized cDNA, 1/25th was mixed with 100 nM each of primer and LightCycler® 480 Sybr Green I master mix (Roche Applied Science) for qPCR analysis. Products were amplified and fluorescent signals acquired with a LightCycler® 480 detection system. The specificity of amplification products was determined by melting curves. UBQ10 was used as internal control for signals normalization. Exor4 relative quantification software (Roche Applied Science) automatically calculated relative expression level of the selected genes with algorithms based on ΔΔCt method. Data were from duplicates of at least two biological replicates. The sequences of primers can be found in Additional file 6: Table SV.

Transcriptomic analyses were performed RNA-seq at the URGV plateform (Evry, France). Three biological replicates were run for each condition.

For GO analyses, the GO Analysis Toolkit and Database for Agriculture Community [55] was used.

### ChIP experiments

ChIP assays were performed on 14-day-old in vitro seedlings using anti-GFP (Santa Cruz), IgG control (Millipore), or anti-H3K9ac (Millipore) antibodies, using a procedure adapted from Gendrel et al. [56]. Briefly, after plant material fixation in 1% (v/v) formaldehyde, tissues were homogenized, nuclei isolated, and lysed. Cross-linked chromatin was sonicated using a water bath Bioruptor UCD-200 (Diagenode, Liège, Belgium) (30 s on/off pulses, at high intensity for 60 min). Protein/DNA complexes were immunoprecipitated with antibodies, overnight at 4 °C with gentle shaking, and incubated for 1 h at 4 °C with 50 μL of Dynabeads Protein A (Invitrogen, Ref. 100-02D). Immunoprecipitated DNA was then recovered using the IPure kit (Diagenode, Liège, Belgium) and analyzed by RT-qPCR. An aliquot of untreated sonicated chromatin was processed in parallel and used as the total input DNA control.

For ChIP-qPCR experiments, fold enrichment of targets in ChIPed DNA relative to input was calculated from an average of three replicate qPCR reactions. The sequences of primers can be found in Additional file 6: Table SV. Positions of the amplified regions on the different loci are indicated in Additional file 1: Figure S30.

### ChIP-seq analysis

After immunoprecipitation of the chromatin, ChIP-seq libraries were generated and sequenced. Alignment was performed using Bowtie [57] v0.12.7 on *Arabidopsis thaliana* genome TAIR10. Default Bowtie parameters were used except for: -best -strata (used to get the best mapping position with the minimum of mismatches). Peak calling was performed with MACS (http://liulab.dfci.harvard.edu/MACS/) [58]. Gene annotation and peak distribution relative to annotated Arabidopsis transcription start site was performed with GPAT [59].

Global clustering of the H3K9ac ChIP-seq data and quantitative comparisons were performed using the seqMINER program (http://bips.u-strasbg.fr/seqminer/) [17]. As reference coordinates, we used the MACS determined peaks for H3K9ac. Tag densities from each ChIP-seq dataset were collected in a window of 1 kb around the reference peak. The collected values were subjected to k-means clustering coupled to linear-based normalization. The normalization procedure reduces bias in the clustering due to inherent differences between ChIP-seq experiments.

### Data availability

RNA-seq and ChIP-seq data from this article were deposited at GEO Project GSE99936.

## Additional files

Additional file 1: Supplemental figures. **Figure S1**–**S5.** HD2B phosphorylation sites. **Figure S6.** MPK3, MPK4, MPK6, and MPK7 antibody specificity. **Figure S7.** MPK3, MPK4, and MPK6 substrate specificity. **Figure S8.** Subcellular localization of MPK3. **Figure S9.** BiFC analysis of HD2B interaction with MPK3, 4, and 6 in epidermal cells of *Agrobacterium*-infiltrated *Nicotiana benthamiana*. **Figure S10.** Co-imunoprecipitation of HD2B with MPK3. **Figure S11.** GO analysis of transcription factors up-regulated in *hd2b* mutant. **Figure S12.** GO analysis of downregulated genes in *hd2b* mutant. **Figure S13.** Validation of transcriptomic data by RT-qPCR. **Figure S14.** Susceptibility of *hd2b* mutant and *HD2B* overexpressing lines. **Figure S15.** Flg22-induced activation of MPK3, MPK4, and MPK6 is similar in Col-0 and *hd2b* mutant. **Figure S16.** Flg22 growth inhibition assay of *hd2b* mutant. **Figure S17.** Flagellin-induced relocalization of HD2B. **Figure S18.** Nuleolar HD2B and nuclear HD2B-ED localization. **Figure S19**. Nuclear HD2B localization. **Figure S20.** Gene expression analyses of *hd2b* and *mpk3* over-expressed genes in the HD2B-AA line. **Figure S21.** Flagellin-independent expression of *HD2B*. **Figure S22.** Specific HD2B targets in mock conditions are involved in defense response. **Figure S23.** Correlation between HD2B binding and gene expression in *hd2b* mutants in mock and flg22 conditions. **Figure S24.** Characterization of H3K9 acetylated regions in mock- and flg22-treated seedlings. **Figure S25.** Comparison between HD2B and H3K9ac peak positions relative to TSS. **Figure S26.** GO analysis of H3K9-hyper and H3K9-hypo acetylated genes after flg22 treatment. **Figure S27.** H3K9 acetylation levels are directly correlated with expression levels after flg22 treatment. **Figure S28.** Gene expression analyses of *hd2b* and *mpk3* flg22 mis-regulated genes in the HD2B-AA line. **Figure S29.** Characterization of *hd2b* mutant line. **Figure S30.** Positions of the regions analyzed by ChIP-qPCR experiments on the different loci. (PDF 1569 kb)

Additional file 2: List of HD2B target genes in mock and flg22 conditions. (XLSX 167 kb)

Additional file 3: List of H3K9 hyper- and hypo-acetylated genes after flg22 treatment. (XLSX 60 kb)

Additional file 4: List of deregulated genes in *hd2b* and *mpk3* mutants. (XLSX 51 kb)

Additional file 5: List of deregulated genes after flg22 treatment in Wt. (XLSX 52 kb)

Additional file 6: List of primers used in this study. (XLSX 13 kb)
