# Supplementary material for: MAPK-triggered chromatin reprogramming by histone deacetylase in plant innate immunity
Source: Genome Biol. 2017 Jul 6;18:131. doi: 10.1186/s13059-017-1261-8 (PMC5501531; doi:10.1186/s13059-017-1261-8)
Supplement: Supplementary file 1 — Supplemental figures. Figure S1–S5. HD2B phosphorylation sites. Figure S6. MPK3, MPK4, MPK6, and MPK7 antibody specificity. Figure S7. MPK3, MPK4, and MPK6 substrate specificity. Figure S8. Subcellular localization of MPK3. Figure S9. BiFC analysis of HD2B interaction with MPK3, 4, and 6 in epidermal cells of Agrobacterium-infiltrated Nicotiana benthamiana. Figure S10. Co-imunoprecipitation of HD2B with MPK3. Figure S11. GO analysis of transcription factors up-regulated in hd2b mutant. Figure S12. GO analysis of downregulated genes in hd2b mutant. Figure S13. Validation of transcriptomic data by RT-qPCR. Figure S14. Susceptibility of hd2b mutant and HD2B overexpressing lines. Figure S15. Flg22-induced activation of MPK3, MPK4, and MPK6 is similar in Col-0 and hd2b mutant. Figure S16. Flg22 growth inhibition assay of hd2b mutant. Figure S17. Flagellin-induced relocalization of HD2B. Figure S18. Nuleolar HD2B and nuclear HD2B-ED localization. Figure S19. Nuclear HD2B localization. Figure S20. Gene expression analyses of hd2b and mpk3 over-expressed genes in the HD2B-AA line. Figure S21. Flagellin-independent expression of HD2B. Figure S22. Specific HD2B targets in mock conditions are involved in defense response. Figure S23. Correlation between HD2B binding and gene expression in hd2b mutants in mock and flg22 conditions. Figure S24. Characterization of H3K9 acetylated regions in mock- and flg22-treated seedlings. Figure S25. Comparison between HD2B and H3K9ac peak positions relative to TSS. Figure S26. GO analysis of H3K9-hyper and H3K9-hypo acetylated genes after flg22 treatment. Figure S27. H3K9 acetylation levels are directly correlated with expression levels after flg22 treatment. Figure S28. Gene expression analyses of hd2b and mpk3 flg22 mis-regulated genes in the HD2B-AA line. Figure S29. Characterization of hd2b mutant line. Figure S30. Positions of the regions analyzed by ChIP-qPCR experiments on the different loci. (PDF 1569 kb) [file 13059_2017_1261_MOESM1_ESM.pdf]

# Additional file 1: Figure S1

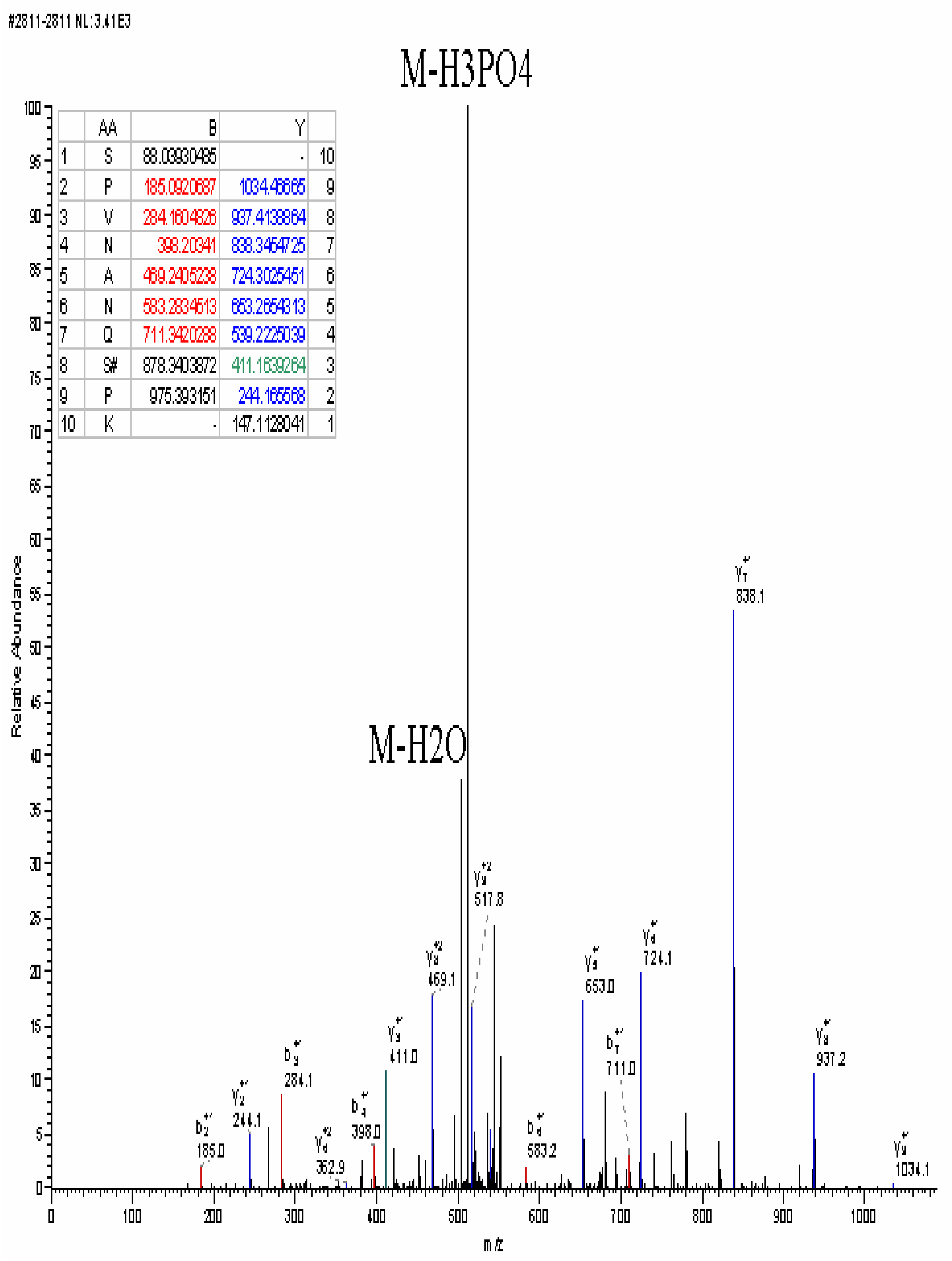


| | AA | B | Y | |
|---|---|---|---|---|
| 1 | S | 88.03930485 | - | 10 |
| 2 | P | 185.0920687 | 1034.46665 | 9 |
| 3 | V | 284.1604826 | 937.4138864 | 8 |
| 4 | N | 398.20341 | 838.3454725 | 7 |
| 5 | A | 469.2405238 | 724.3025451 | 6 |
| 6 | N | 583.2834513 | 653.2654313 | 5 |
| 7 | Q | 711.3420288 | 539.2225039 | 4 |
| 8 | S# | 878.3403872 | 411.1639264 | 3 |
| 9 | P | 975.393151 | 244.165568 | 2 |
| 10 | K | - | 147.1128041 | 1 |

**Figure S1: HD2B phosphorylation sites.**
Mass spectra of IMAC-purified phosphopeptides of HD2B upon phosphorylation by MPK3 [13].

# Additional file 1: Figure S2

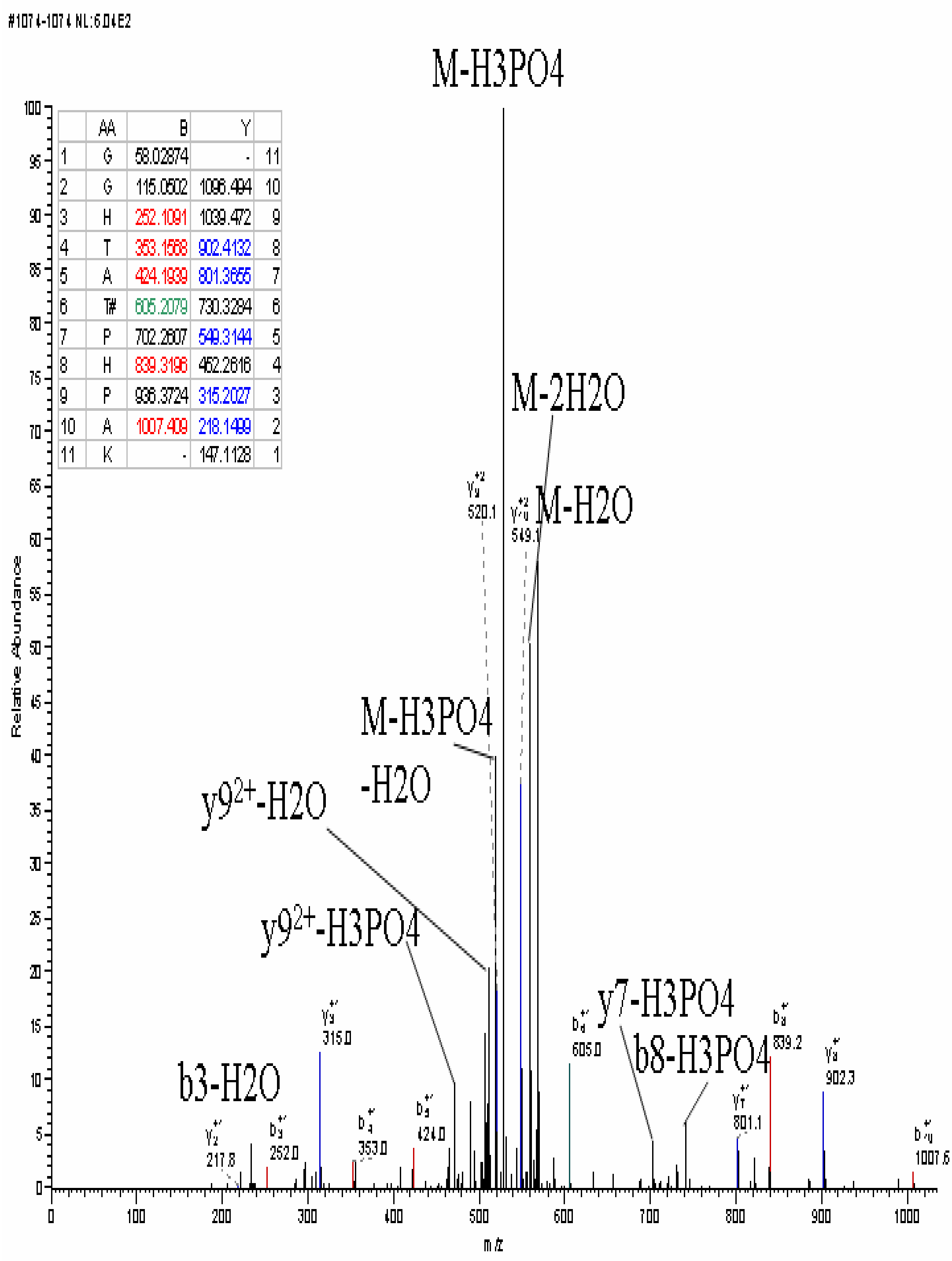


**Figure S2: HD2B phosphorylation sites.**

Mass spectra of IMAC-purified phosphopeptides of HD2B upon phosphorylation by MPK3 [13].

# Additional file 1: Figure S3

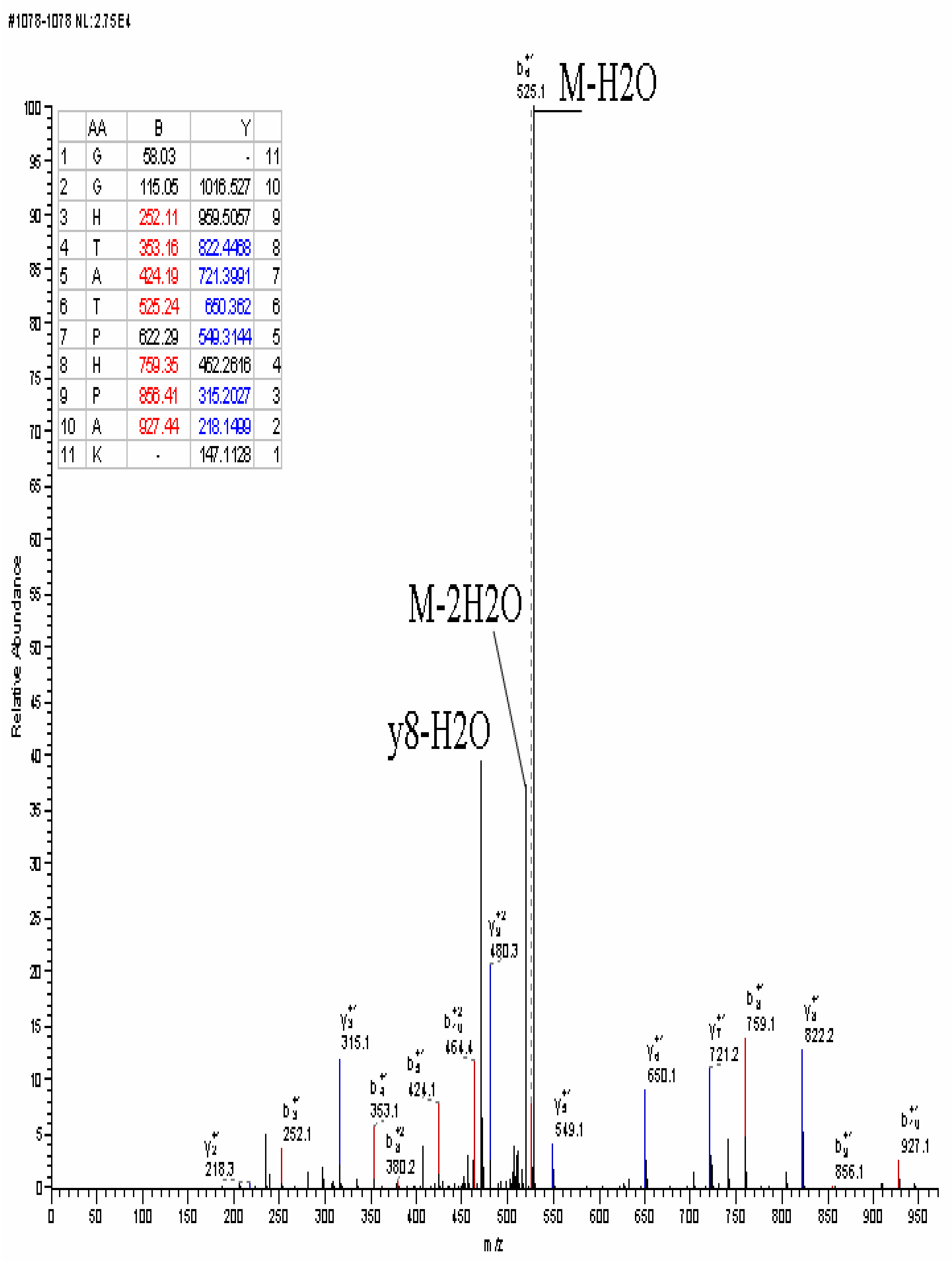


**Figure S3: HD2B phosphorylation sites.**
Mass spectra of IMAC-purified phosphopeptides of HD2B upon phosphorylation by MPK3 [13].

# Additional file 1: Figure S4

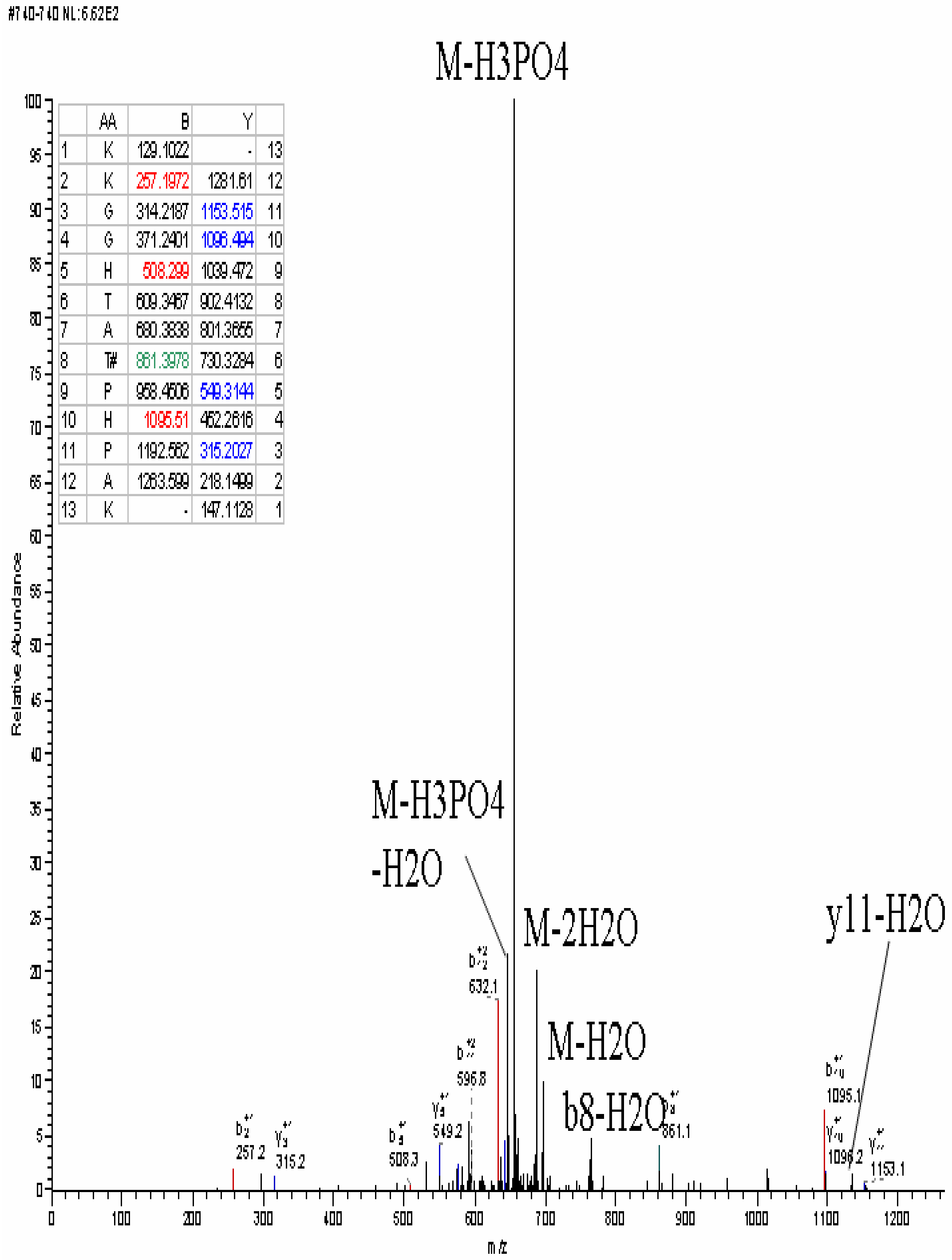


| | AA | B | Y | |
|---|---|---|---|---|
| 1 | K | 129.1022 | - | 13 |
| 2 | K | 257.1972 | 1281.61 | 12 |
| 3 | G | 314.2187 | 1153.515 | 11 |
| 4 | G | 371.2401 | 1096.494 | 10 |
| 5 | H | 508.299 | 1039.472 | 9 |
| 6 | T | 609.3467 | 902.4132 | 8 |
| 7 | A | 680.3838 | 801.3655 | 7 |
| 8 | T# | 861.3978 | 730.3284 | 6 |
| 9 | P | 958.4506 | 549.3144 | 5 |
| 10 | H | 1095.51 | 452.2616 | 4 |
| 11 | P | 1192.562 | 315.2027 | 3 |
| 12 | A | 1263.599 | 218.1499 | 2 |
| 13 | K | - | 147.1128 | 1 |

**Figure S4: HD2B phosphorylation sites.**
Mass spectra of IMAC-purified phosphopeptides of HD2B upon phosphorylation by MPK3 [13].

# Additional file 1: Figure S5

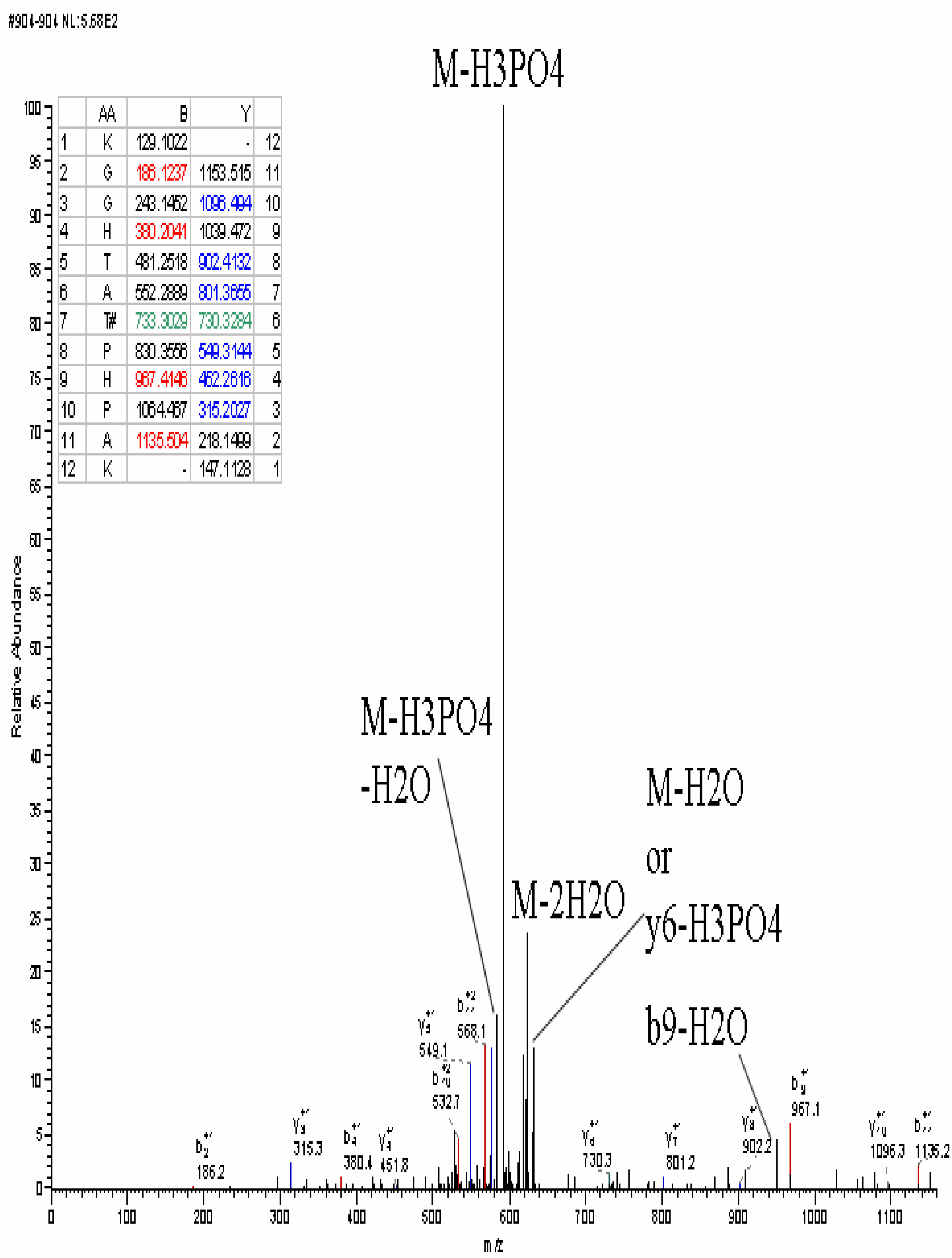


| | AA | B | Y | |
|---|---|---|---|---|
| 1 | K | 129.1022 | - | 12 |
| 2 | G | 186.1237 | 1153.515 | 11 |
| 3 | G | 243.1452 | 1096.494 | 10 |
| 4 | H | 380.2041 | 1039.472 | 9 |
| 5 | T | 481.2518 | 902.4132 | 8 |
| 6 | A | 552.2889 | 801.3655 | 7 |
| 7 | T# | 733.3029 | 730.3284 | 6 |
| 8 | P | 830.3556 | 549.3144 | 5 |
| 9 | H | 967.4146 | 452.2616 | 4 |
| 10 | P | 1064.467 | 315.2027 | 3 |
| 11 | A | 1135.504 | 218.1499 | 2 |
| 12 | K | - | 147.1128 | 1 |

**Figure S5: HD2B phosphorylation sites.**
Mass spectra of IMAC-purified phosphopeptides of HD2B upon phosphorylation by MPK3 [13].

# Additional file 1: Figure S6

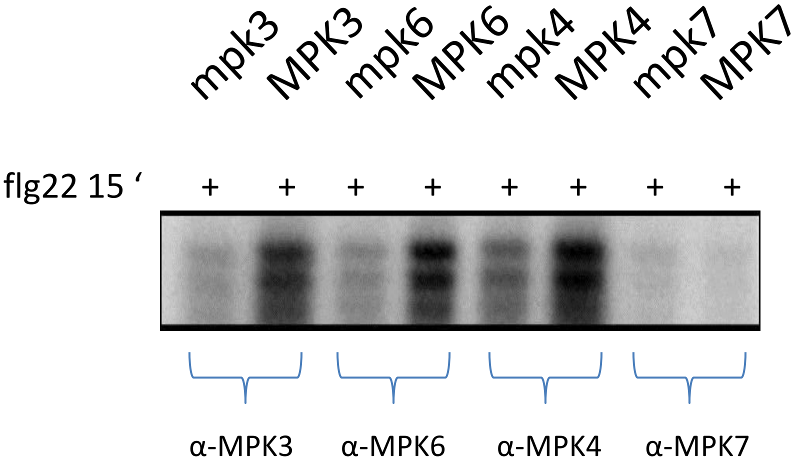


**Figure S6: MPK3, MPK4, MPK6 and MPK7 antibody specificity.**
*In vitro* phosphorylation assays of MBP by MPK3, MPK4, MPK6 and MPK7. MPK3, 4, 6 and 7 were immuno-precipitated from flagellin-treated 14 d old plants of *mpk3, MPK3, mpk4, MPK4, mpk6, MPK6, mpk7* and *MPK7.* MPK3, 4 and 6 were able to phosphorylate MBP. MPK7 served as a negative control.

# Additional file 1: Figure S7

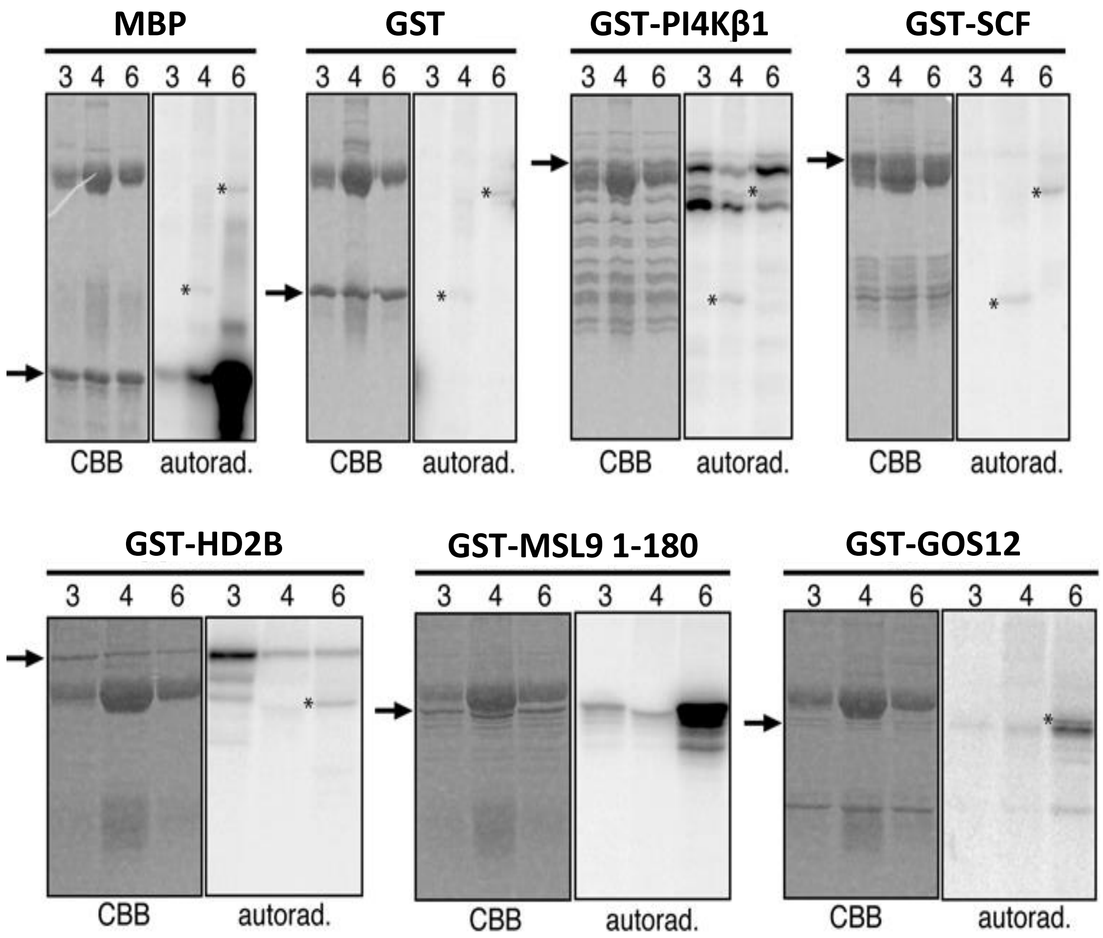


**Figure S7: MPK3, MPK4 and MPK6 substrate specificity.**

*In vitro* phosphorylation assays of putative MAPK substrates by MPK3 (3), MPK4 (4) and MPK6 (6). MPK3, 4 and 6 were immuno-precipitated from flagellin-treated root cell suspensions. For each assay, Coomassie staining of the gel (CBB) is shown on the left as a protein loading control and the autoradiography (Autorad) is shown on the right. The following putative MAPK substrates were tested for MPK3, MPK4 and MPK6 substrate specificity: HDT2 (At5g22650), MSL9 (At5g19520), GOS12 (At2g45200), PGI1 (At4g24620), PI-4Kß1 (At5g64070), SCF (At5g13300).

# Additional file 1: Figure S8

**A.** - 2nd biological replicate of experiment figure 1C :

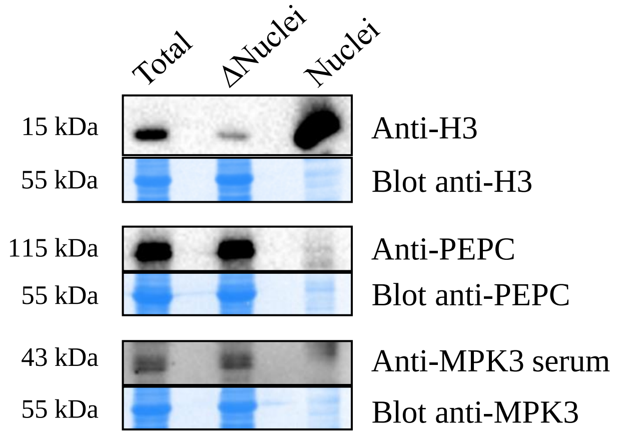


- 3rd biological replicate of experiment figure 1C :

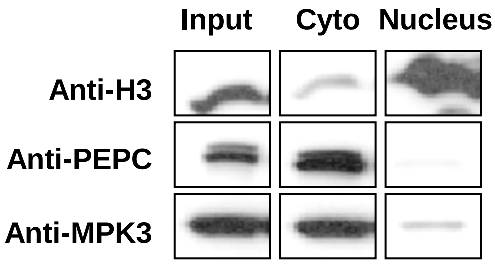


**B.**

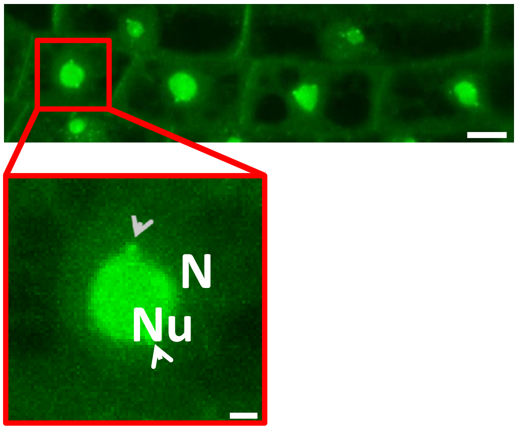


**Figure S8: Subcellular localization of MPK3**

A: Biological replicates of cell fractionation assay of MPK3 localization. Whole cell extracts (Total input) and proteins extracted from the cytoplasmic and the nuclear fractions were subjected to SDS-PAGE and analyzed by immunoblot. Anti-H3 and anti-PEPC antibodies were used as controls for nuclear and cytoplasmic proteins, respectively.

B: Preferential localization of GFP-MPK3 in nucleus (N), nucleolus (Nu) and perinucleolar compartments (arrows). Localization in cytoplasm is rather weak. Bars represent 10 µm for upper image and 2 µm for lower detailed image.

# Additional file 1: Figure S9

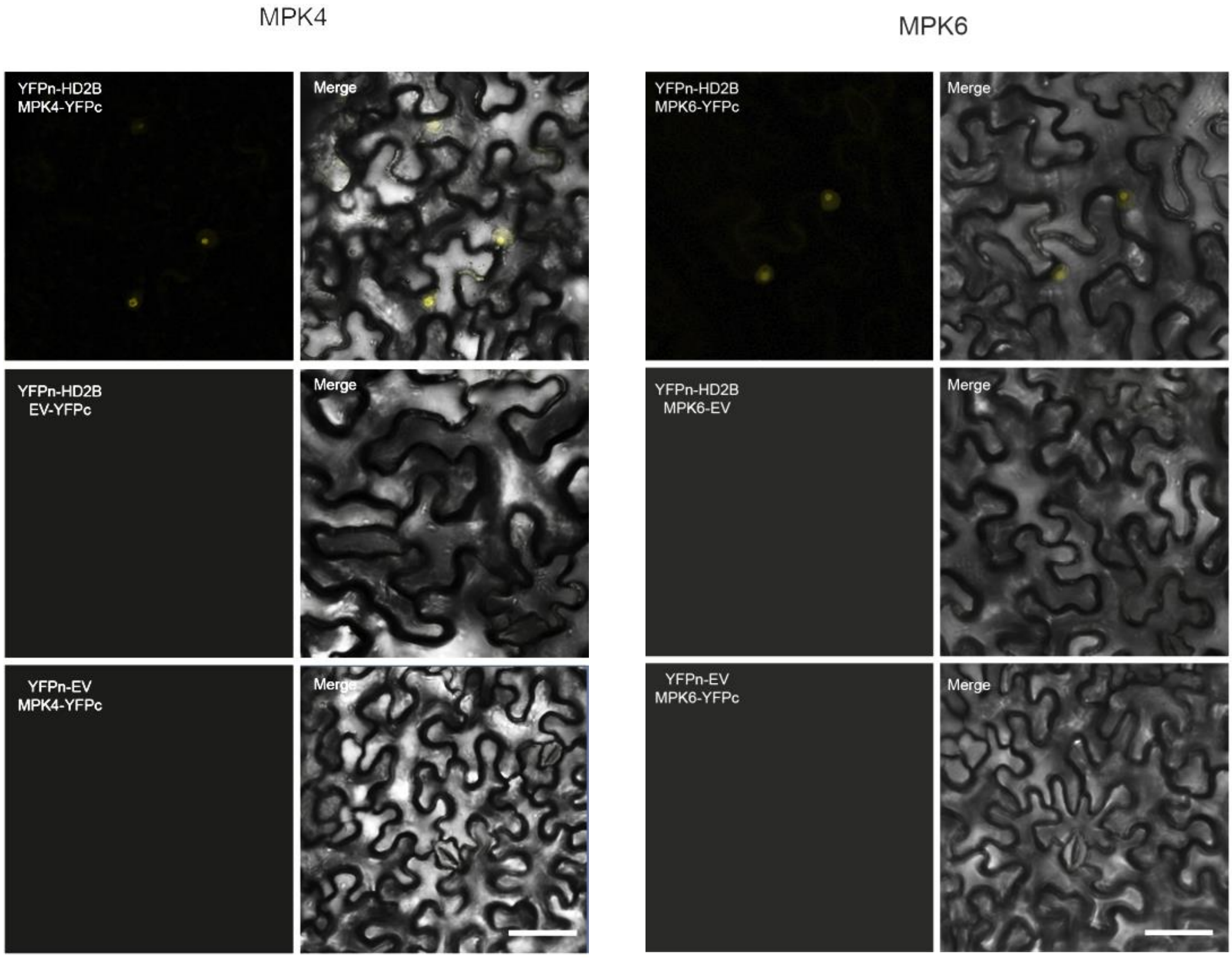


**Figure S9: BiFC analysis of HD2B interaction with MPK3, 4 and 6 in epidermal cells of *Agrobacterium*-infiltrated *Nicotiana benthamiana*.**

Empty vectors were used as controls. Fluorescence indicates that YFPn-HD2B interacts with MPK3, 4 and 6-YFPc. YFPn-HD2B does not interact with empty YFPc vector and MPK3, 4 and 6-YFPc does not interact with empty YFPn vector. Right panels indicate merging the YFP and light transmission images.

# Additional file 1: Figure S10

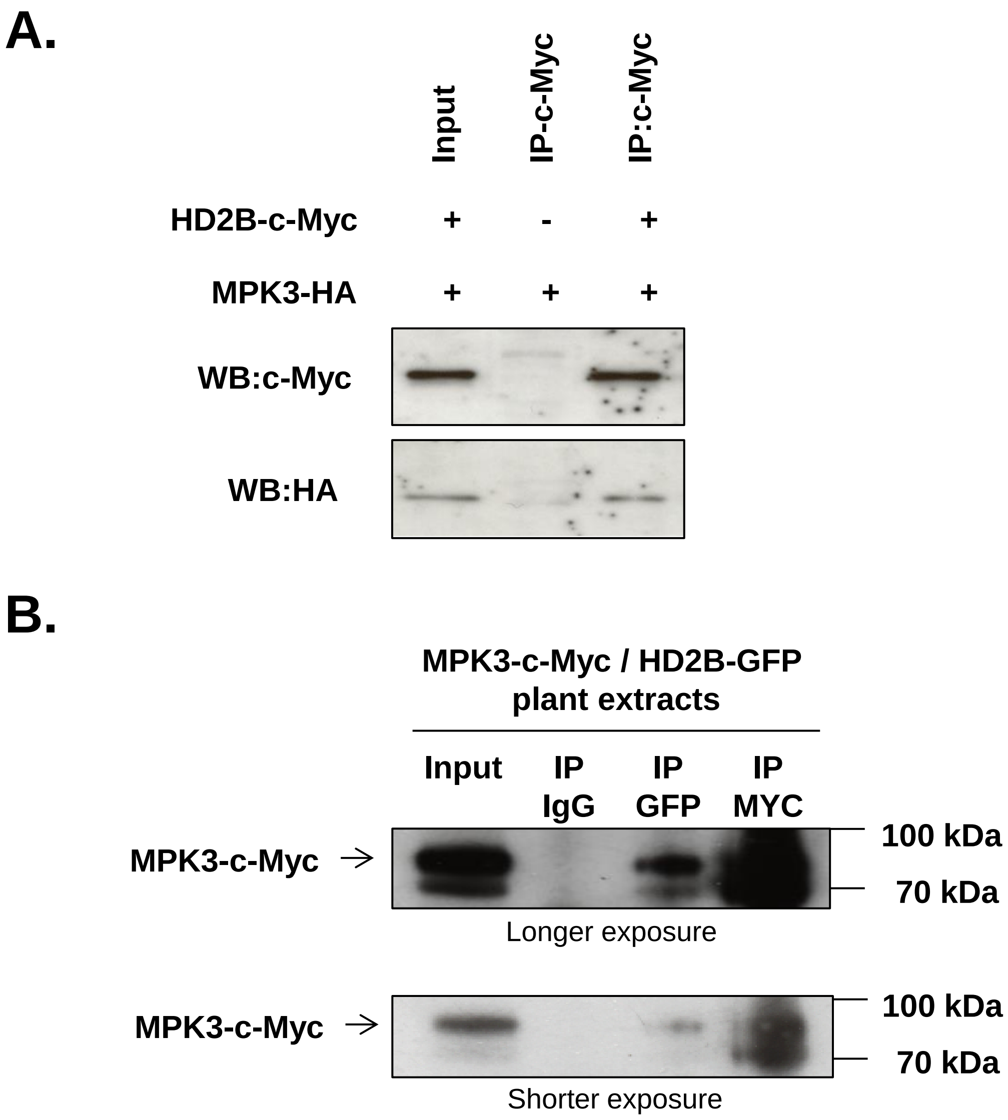


**Figure S10: Co-imunoprecipitation of HD2B with MPK3**

A: Proteins extracted from protoplasts transiently expressing HD2B-c-Myc together with either MPK3-HA or MPK6-HA were immuno-precipitated with an anti-HA antibody. After SDS-PAGE, an immunoblot was performed using an anti-HA or an anti-c-Myc antibody. MPK3 but not MPK6 was immunoprecipitated with HD2B.

B: H2DB interacts with MPK3 *in planta*. Proteins extracted from plants expressing MPK3-c-Myc and GFP-HD2B were immunoprecipitated with IgG, anti GFP or anti-c-Myc antibodies, separated by SDS-PAGE and revealed by immunoblotting using an anti-c-Myc antibody. MPK3-c-Myc was detected in the input and after IP using anti-GFP or anti-c-Myc antibodies, but not after IP using the control IgG.

# Additional file 1: Figure S11

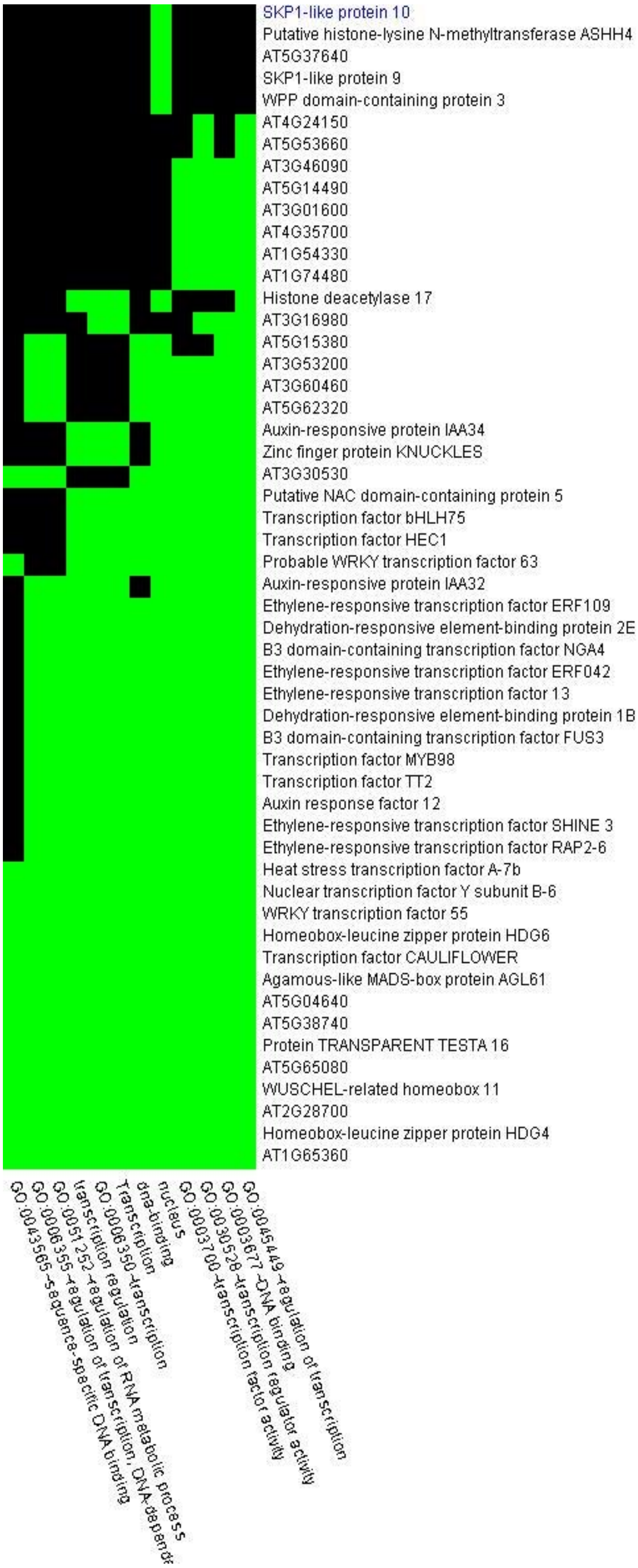


**Figure S11: GO analysis of transcription factors up-regulated in *hd2b* mutant.**
Green and black squares correspond respectively to a positive or a negative gene ontology association.

# Additional file 1: Figure S12

A.

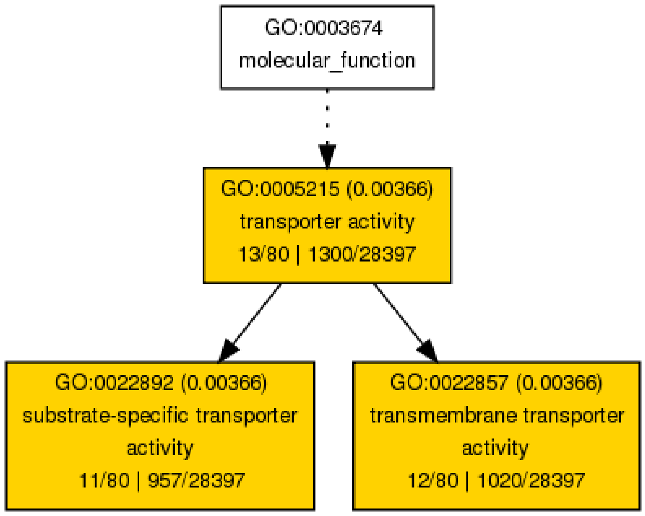


B.

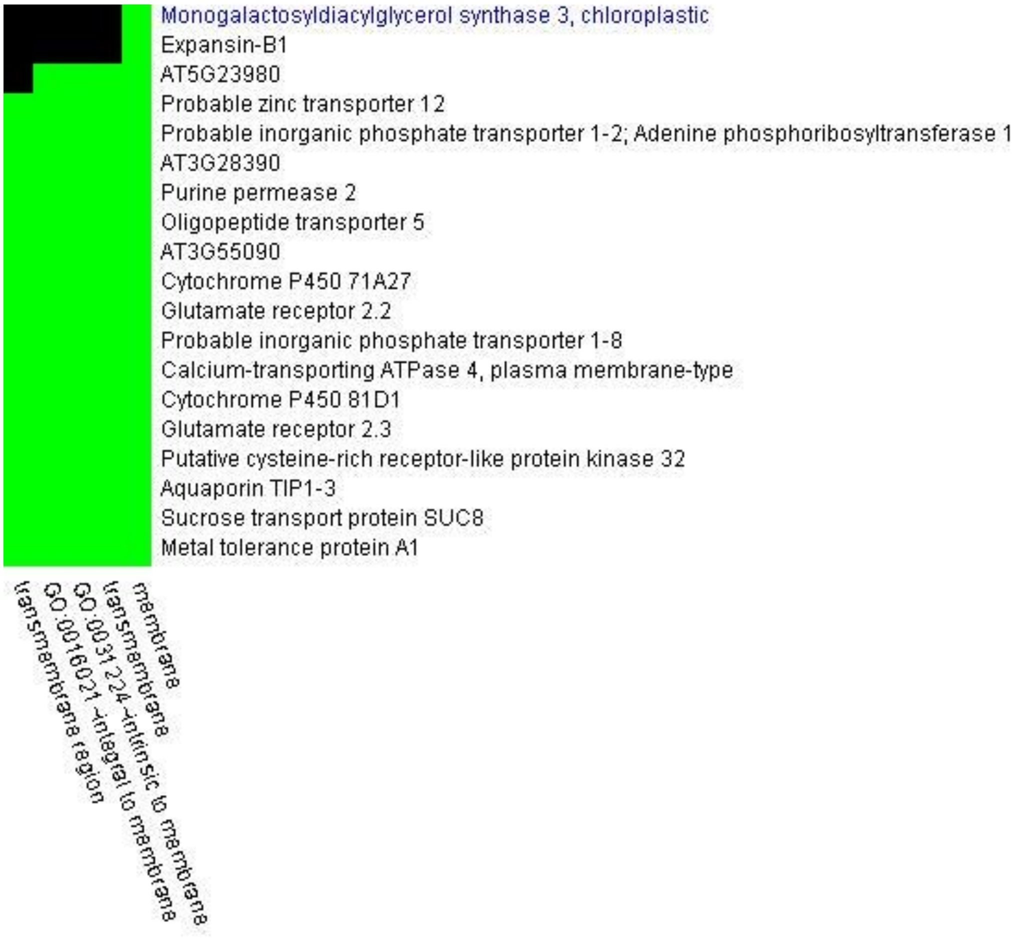


**Figure S12: GO analysis of down-regulated genes in *hd2b* mutant.**
A: GO terms emanating from the analysis of down-regulated genes in hd2b mutants.
B: Down-regulated gene list corresponding to the GO terms indicated in S5A.

## Additional file 1: Figure S13

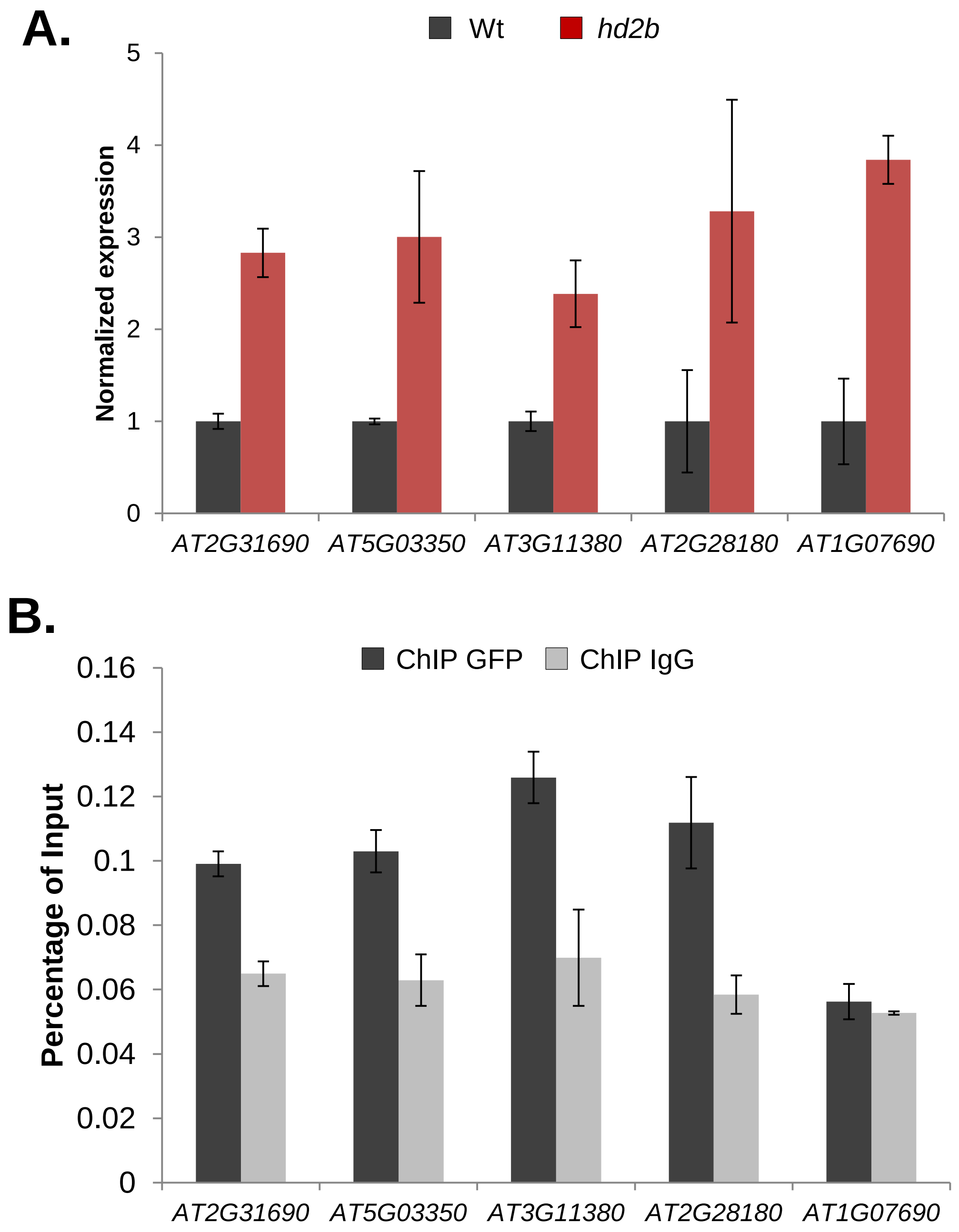


**Figure S13: Validation of transcriptomic data by RT-qPCR**.

A: The up-regulation of 5 genes, selected among the 150 commonly up-regulated genes in *hd2b* and *mpk3* mutants and flg22-induced in wild type plants, was confirmed by RT-qPCR in *hd2b* and *mpk3* mutants.

B: HD2B protein binding on flg22 inducible-genes in mock conditions. ChIP-qPCR assays with anti-GFP antibodies were performed on *pHD2B::GFP-HD2B* seedlings using oligonucleotides in the proximal promoter region of the 5 selected loci. IgG antibody was used as a negative control. An enrichment of GFP-HD2B protein is observed on *AT2G31690*, *AT5G03350*, *AT3G11380* and *AT2G28180* but not on *AT1G07690*, which is consistent with HD2B ChIP-seq data.

# Additional file 1: Figure S14

## Susceptibility of *hd2b* and *HD2B* overexpressing lines to *Pst DC3000* infection

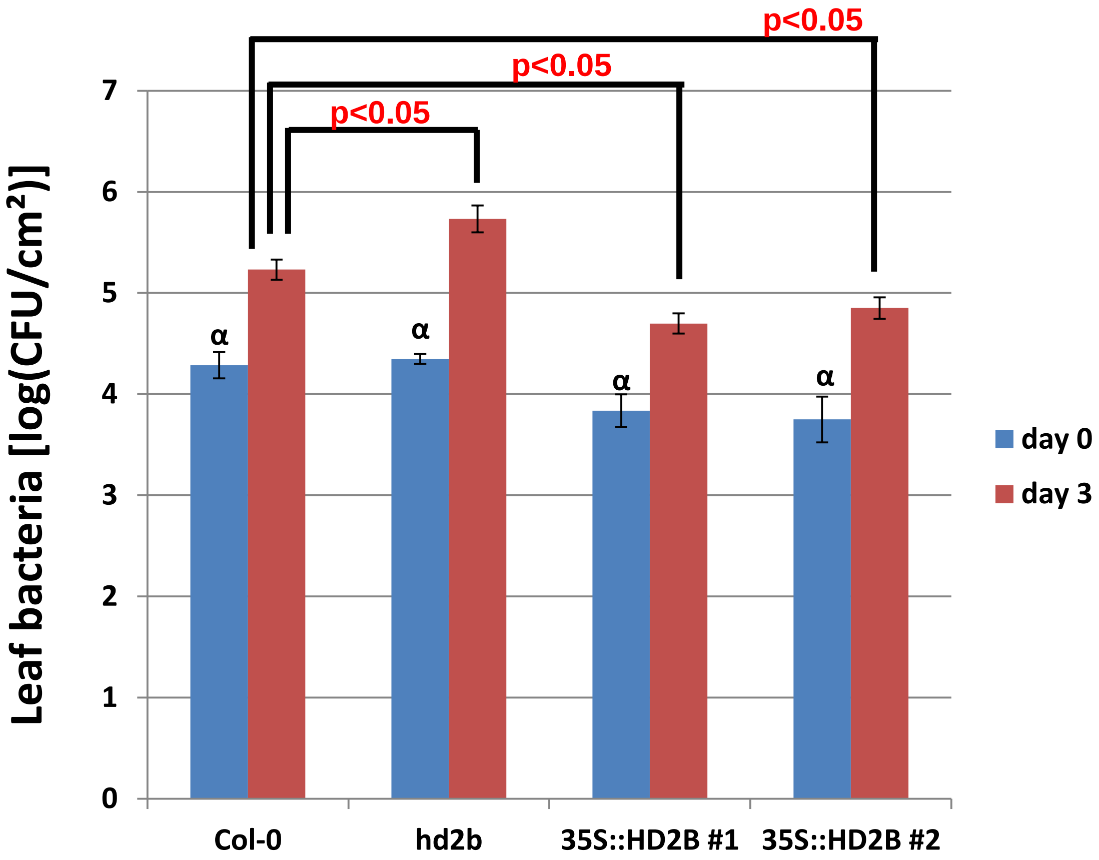


**Figure S14: Susceptibility of *hd2b* mutant and *HD2B* overexpressing lines**. *35S::HD2B* susceptibility to virulent *Pseudomonas syringae* pv. tomato PstDC3000 was compared to wild-type col-0 plants. Leaves were infiltrated with a suspension of PstDc3000, and bacteria were quantified 2 hrs after inoculation (day 0) and three days later (day 3). Mutant plants showed enhanced susceptibility as evidenced by the increased bacterial growth, whereas HD2B over-expressing lines were more resistant to PstDC3000. Data are from three independent experiments, values are averaged and standard deviations are shown. For each condition (day 0, day 3), a one way ANOVA followed by an all pairwise multiple comparison procedure was performed (Holm-Sidak method, 23>n>8, $p<0.05$).

# Additional file 1: Figure S15

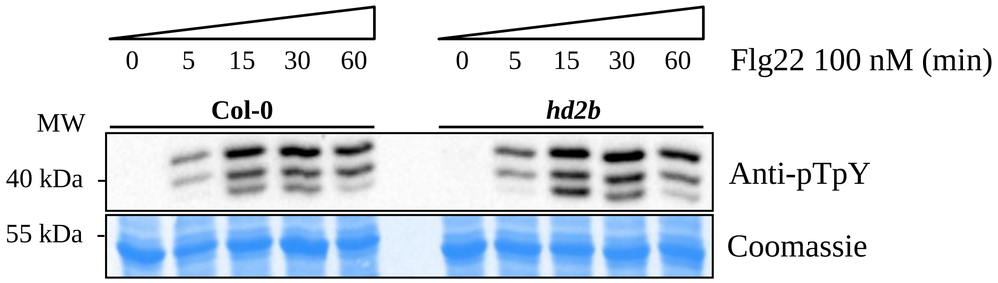


**Figure S15: Flg22-induced activation of MPK3, MPK4 and MPK6 is similar in Col-0 and *hd2b* mutant.**

Immunoblotting analyses were realized with Col-0 and *hd2b* mutant at the indicated time-points after 100 nM flg22 treatment for 0, 5, 15, 30 and 60 min, using an anti-pTpY antibody that detects activated MPK3, MPK4 and MPK6. Blots were stained with Coomassie blue for protein visualization, and the protein band corresponding to the RuBisCO large subunit is shown. MW stands for molecular weight.

# Additional file 1: Figure S16

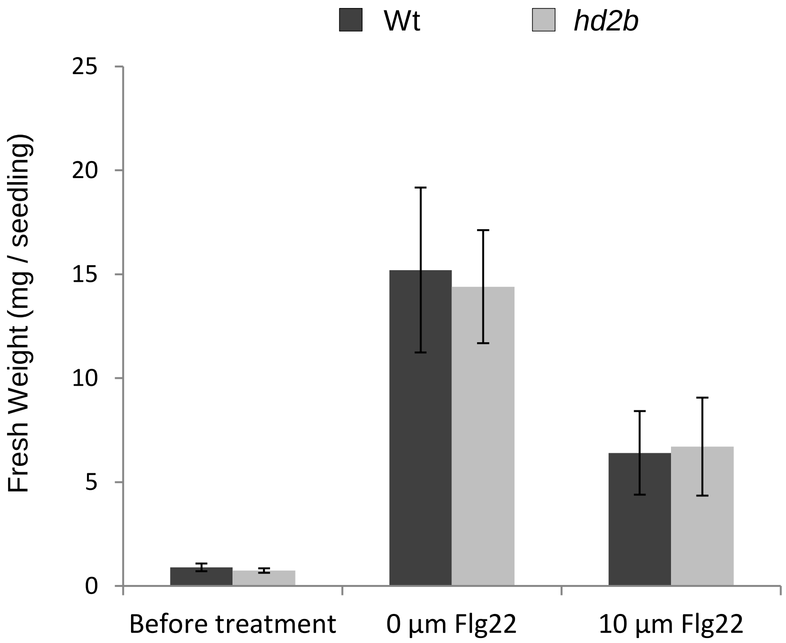


**Figure S16: Flg22 growth inhibition assay of *hd2b* mutant.**

Wt and *hd2b* mutant seedlings were grown for 6 days and transferred to a medium in the absence or presence of 10 µm of flg22. Fresh weight of 10 seedlings was measured before and after the treatment.

# Additional file 1: Figure S17

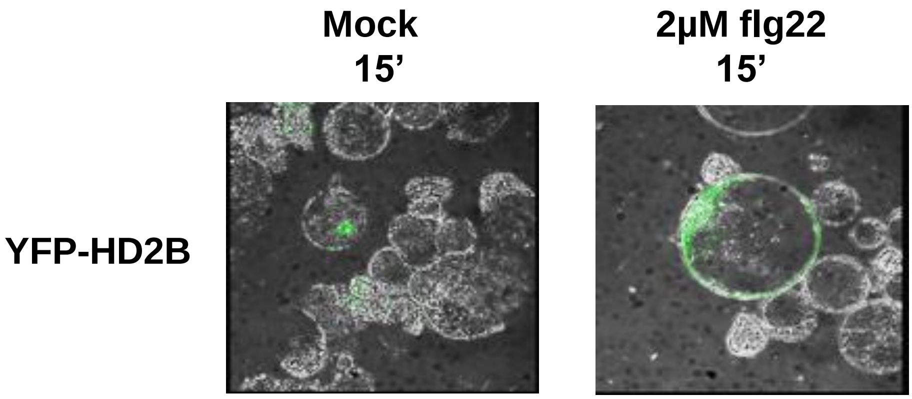


**Figure S17: Flagellin-induced relocalization of HD2B.**
HD2B relocalizes from the nucleolus to the nucleoplasm of protoplasts after flagellin treatment. HD2B-YFP was expressed in protoplasts that were either mock treated or treated with 2µM of flg22 for 15 minutes. HD2B-YFP is strongly present in the nucleolus in mock conditions whereas the protein accumulates in the whole nucleus after flg22 treatment.

# Additional file 1: Figure S18

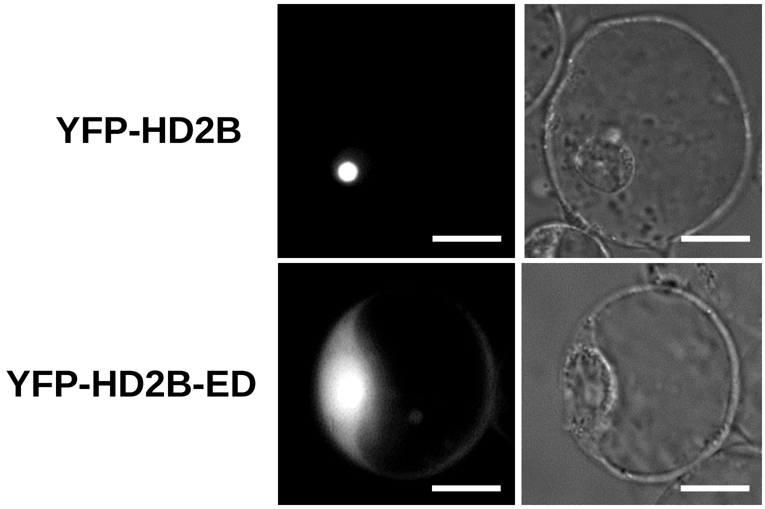


**Figure S18: Nuleolar HD2B and nuclear HD2B-ED localization.**
A mutated form of YFP-HD2B that mimics a constitutively phosphorylated version of the HD2B protein (HD2B-ED) was expressed in Arabidopsis protoplast cells. The wild type YFP-HD2B form was localized in the nucleolus while the mutant YFP-HD2B-ED form accumulated throughout the nucleus. Scale bars, 20µm.

# Additional file 1: Figure S19

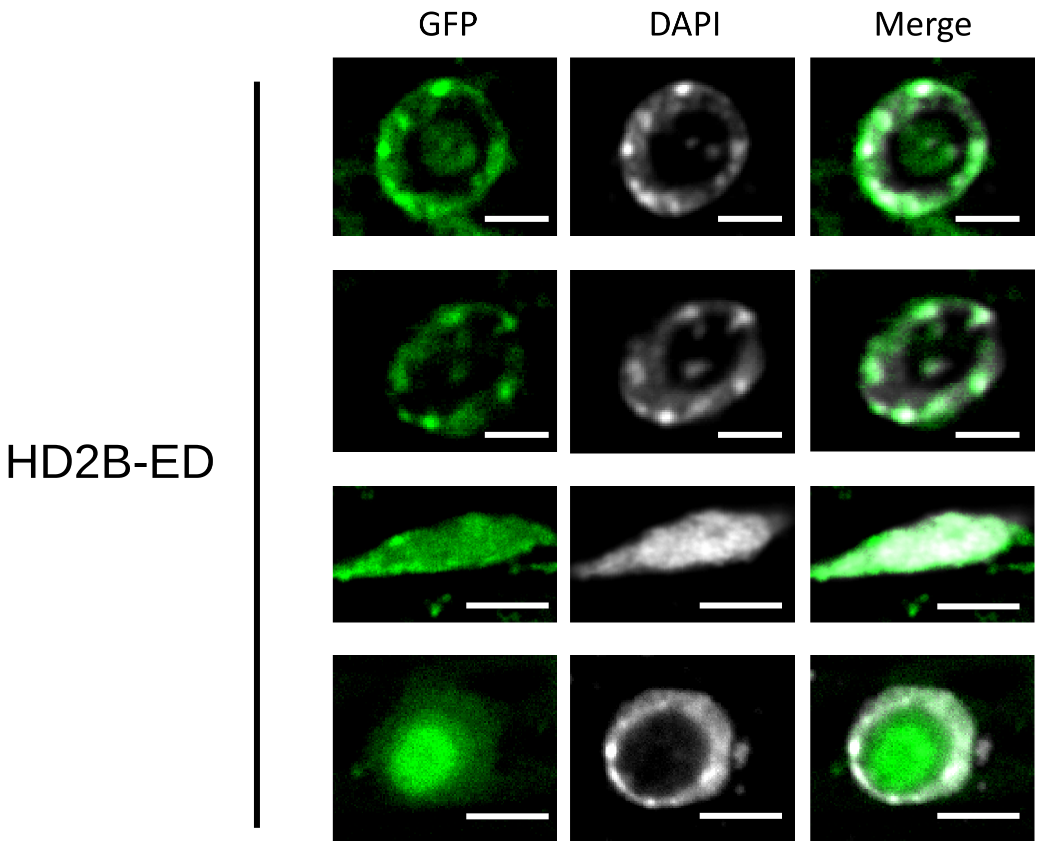


**Figure S19: Nuclear HD2B localization.**
GFP-HD2B-ED that mimics a constitutively phosphorylated version of HD2B was stably expressed in Arabidopsis plants and accumulated in the nucleoplasm. The relative presence of the protein in the nucleolus and the nucleoplasm varies among the observed nuclei. In all cases, signal was observed outside of the nucleolus although the protein was not systematically excluded from this compartment. Scale bars, 5µm.

# Additional file 1: Figure S20

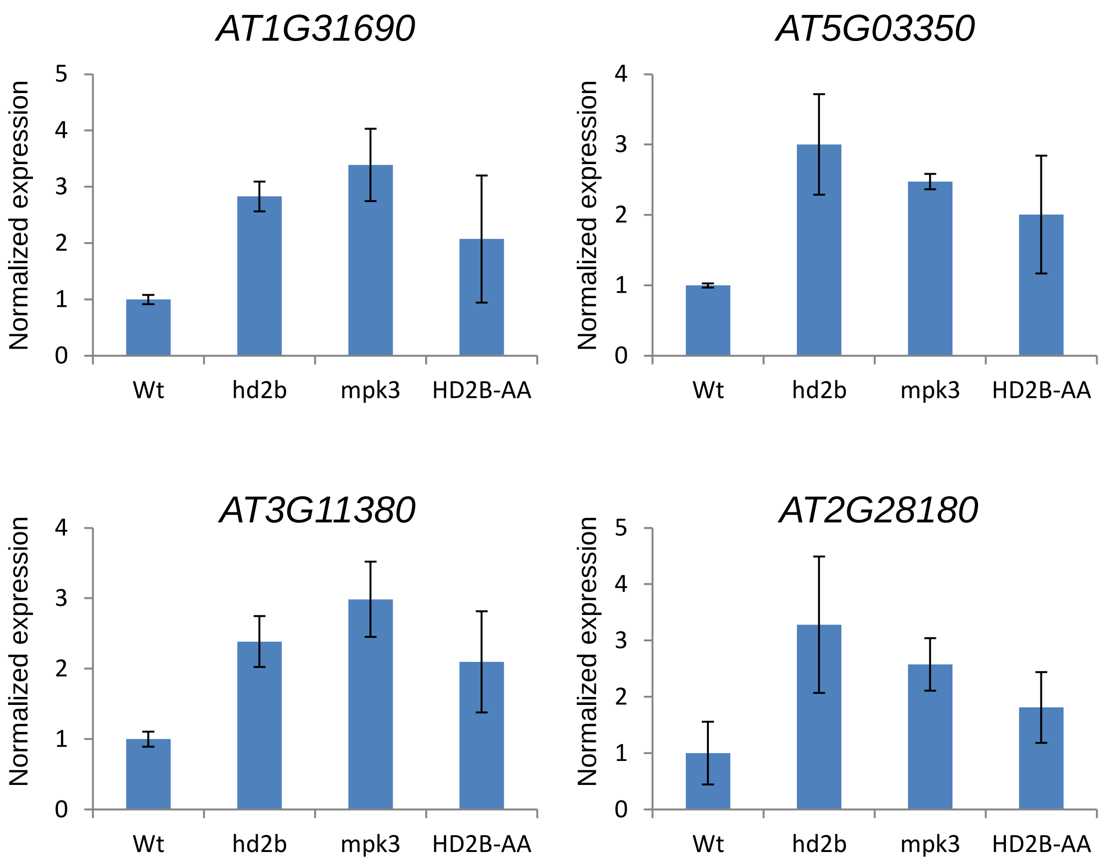


**Figure S20: Gene expression analyses of *hd2b* and *mpk3* over-expressed genes in the HD2B-AA line.**

# Additional file 1: Figure S21

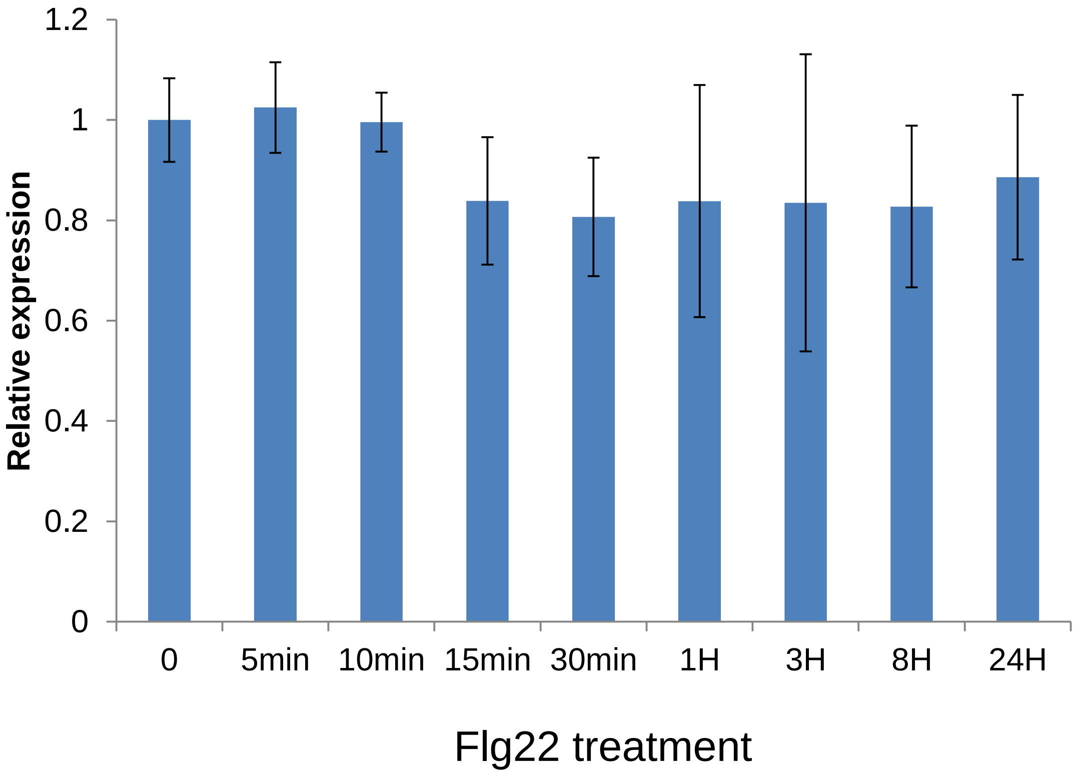


**Figure S21: Flagellin-independent expression of *HD2B*.**
HD2B expression was determined by qPCR at the given times before and after flg22 treatment of col-0 plants.

# Additional file 1: Figure S22

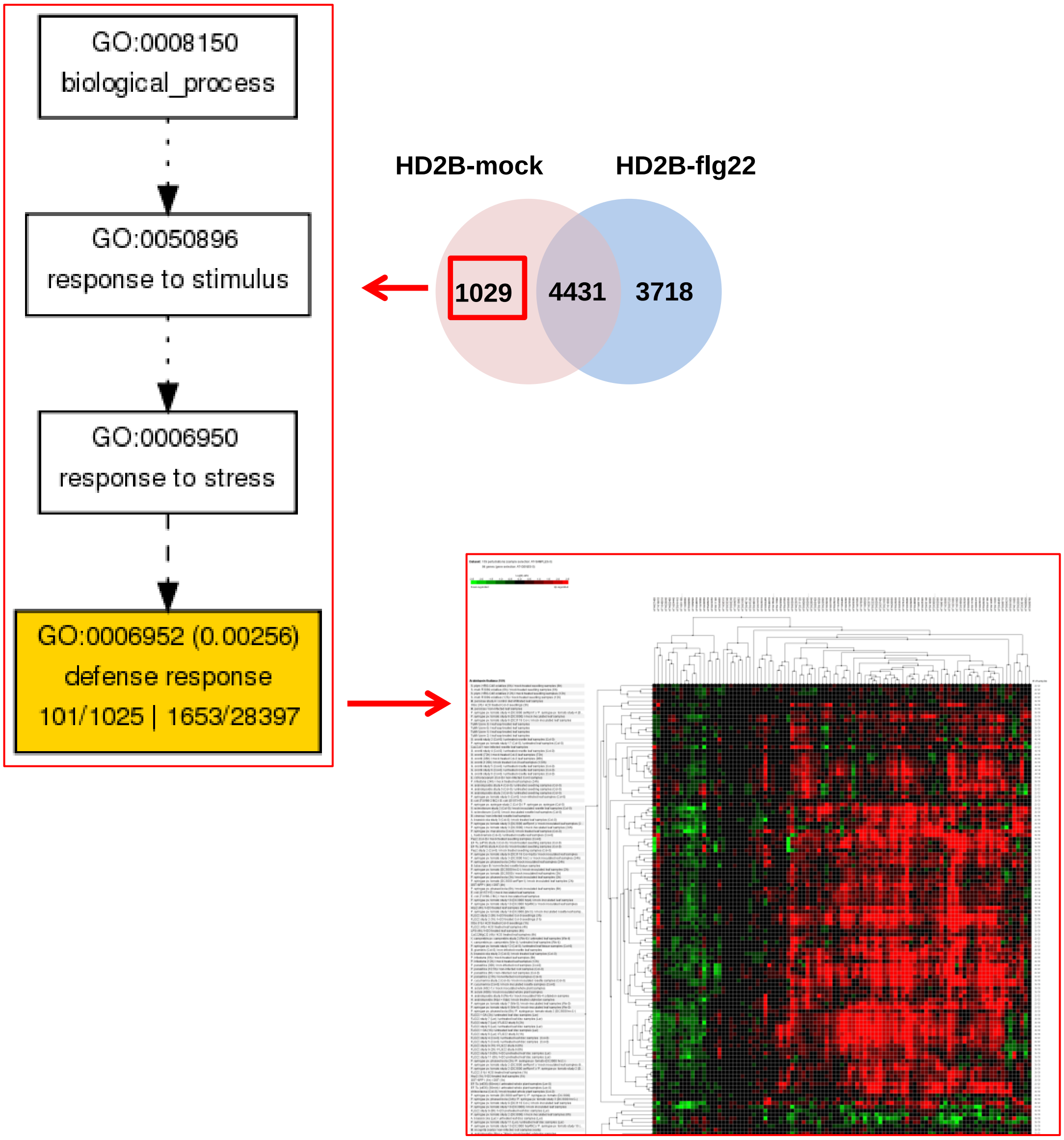


**Figure S22: Specific HD2B targets in mock conditions are involved in defense response**. Venn diagram represents overlap between HD2B targets in mock conditions and in flg22 conditions. The hierarchical tree graph generated with Agrigo GO Analysis Toolkit (*left* shows a significant enrichment in “defense response”of the specific HD2B targets under mock conditions). The heatmap generated with public available microarrays data and Genevestigator software 56 indicates that specific mock-HD2B-target genes are induced after biotic or elicitor stresses. Each column represents a gene from the list, and each line represents a particular microarray experiment. Red, green and black colors indicate an up-regulation, a down-regulation, and no change of gene expression, respectively (*right*).

# Additional file 1: Figure S23

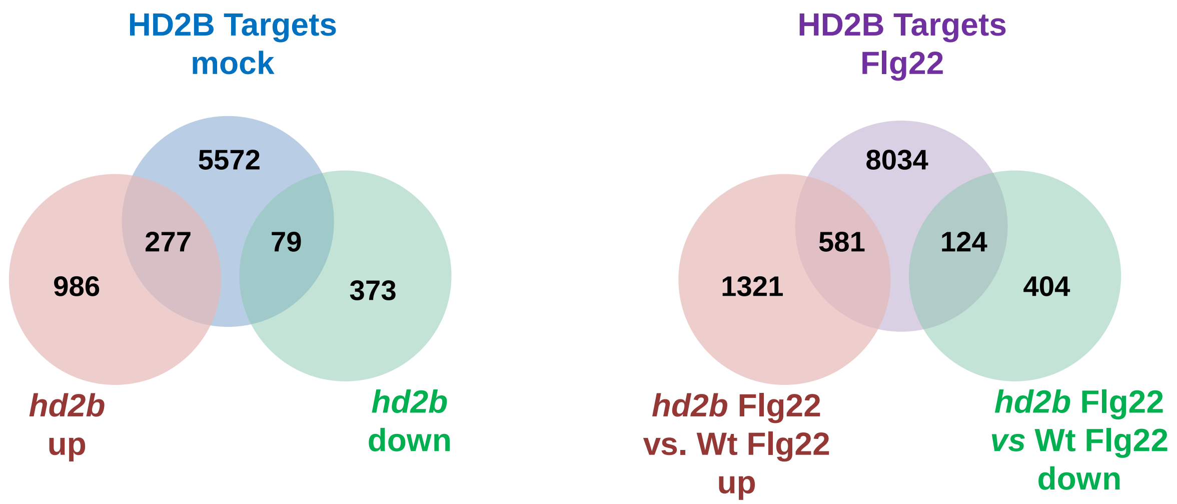


**Figure S23: Correlation between HD2B binding and gene expression in *hd2b* mutants in mock and flg22 conditions.**

# Additional file 1: Figure S24

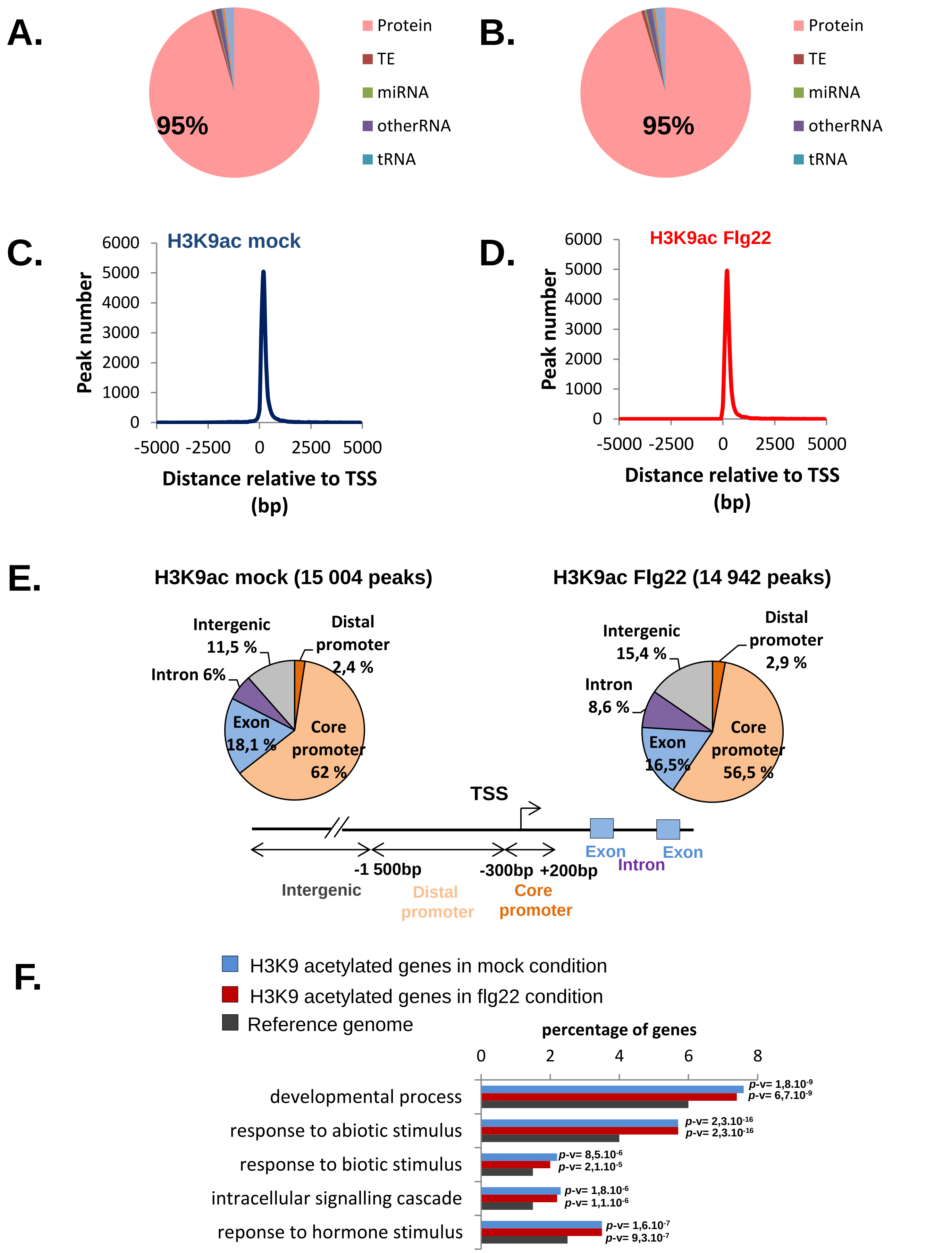

**Figure S24: Characterization of H3K9 acetylated regions in mock- and flg22-treated seedlings.**

**A-B**: The majority of H3K9 acetylated genes are protein coding genes. The H3K9 acetylated

genes of WT seedlings either mock (A) or flg22-treated (B) for 30 min were determined by ChIP-seq experiments using an anti-H3K9ac antibody. The genomic annotation of H3K9ac peaks was performed using the Genomic Position Annotation Tool (GPAT).

**C-D**: Most of H3K9ac peaks are around 300 bp downstream of the TSS. No obvious differences in H3K9ac peak distributions were observed between mock (C) and flg22 (D) treated plants.

**E**: Most H3K9ac peaks are present in the core promoter of genes. Core promoters were defined as the region between -300 and +200 bp relative to the TSS, while the distal promoter was defined between -1500 and -300 bp relative to the TSS. Other genomic regions represent intergenic regions, excluding exons and introns.

**F**: GO analysis of H3K9 acetylated genes. GO data were extracted with the Agrigo GO Analysis Toolkit. A histogram highlights the enrichment of the GO classes. P-values for each enriched class are indicated (*p*-v).

# Additional file 1: Figure S25

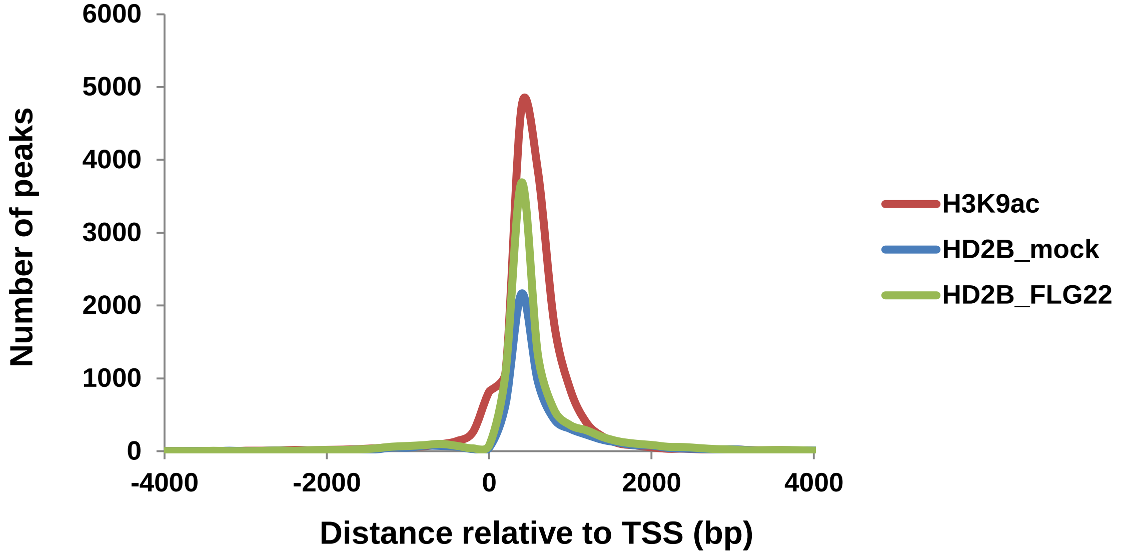


**Figure S25: Comparison between HD2B and H3K9ac peak positions relative to TSS.**

# Additional file 1: Figure S26

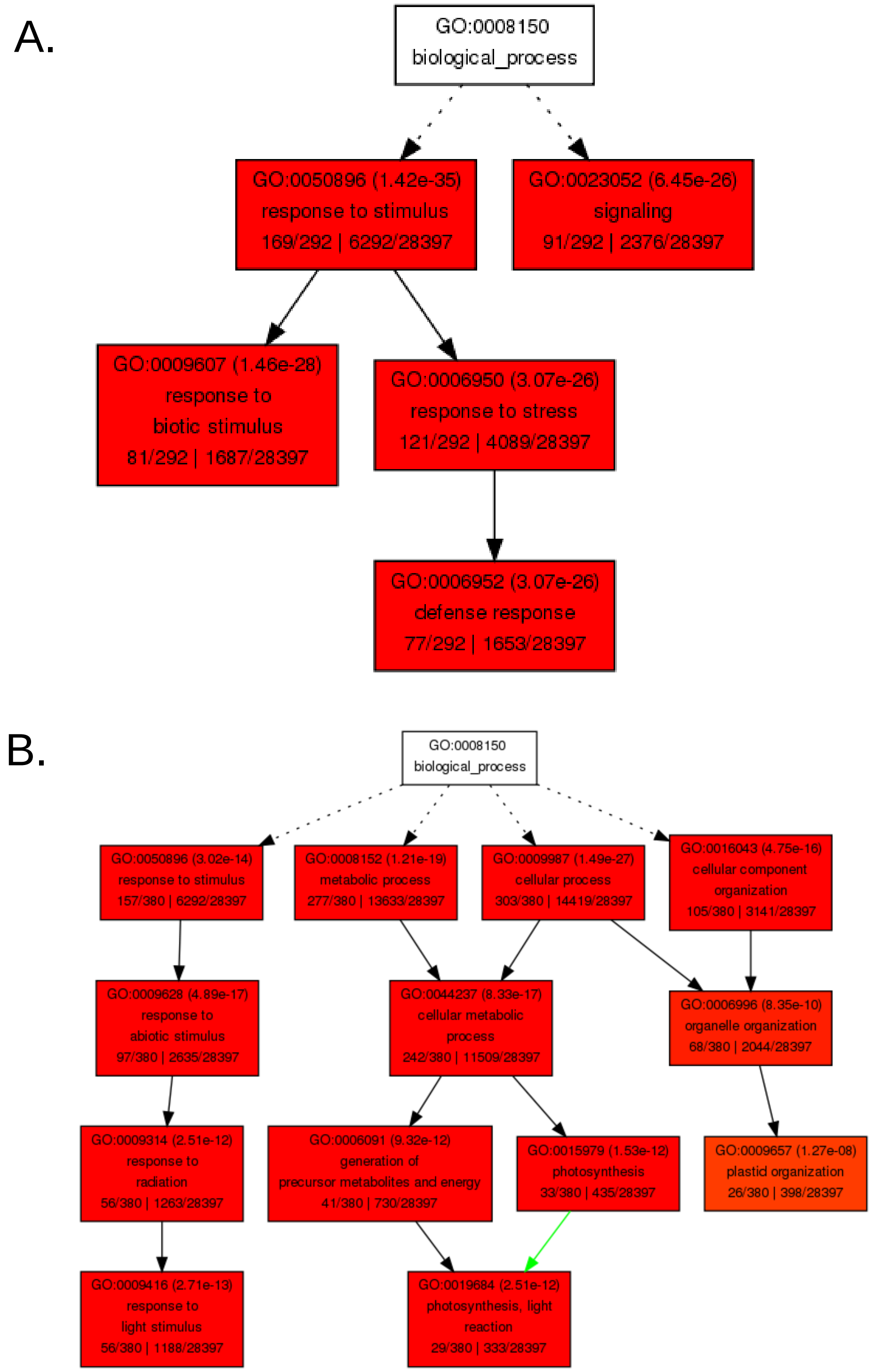


**Figure S26: GO analysis of H3K9-hyper and H3K9-hypo acetylated genes after flg22 treatment**.

Using the Agrigo GO Analysis Toolkit, a GO analysis was performed for H3K9-hyper acetlated (A) and H3K9-hypo acetylated (B) genes after flg22 treatment.

# Additional file 1: Figure S27

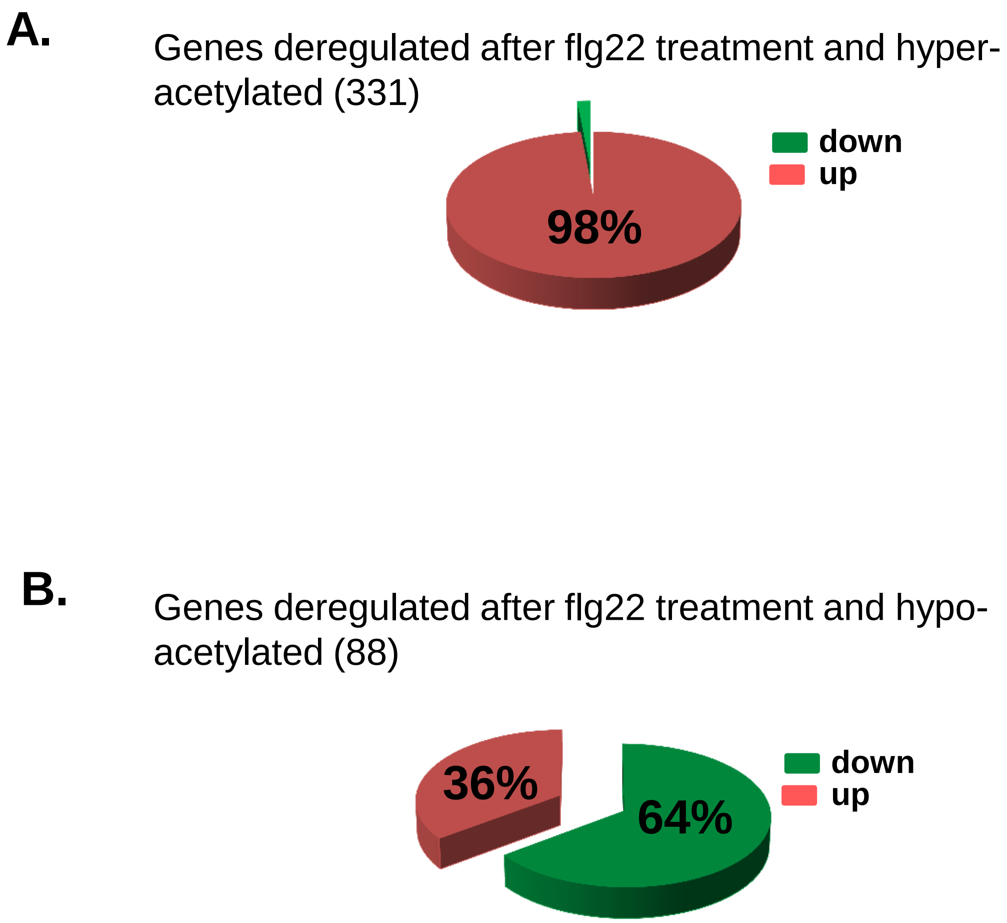


**Figure S27: H3K9 acetylation levels are directly correlated with expression levels after flg22 treatment**.
Most H3K9 hyper-acetylated genes are activated (A) while H3K9 hypo-acetylated genes are repressed (B).

# Additional file 1: Figure S28

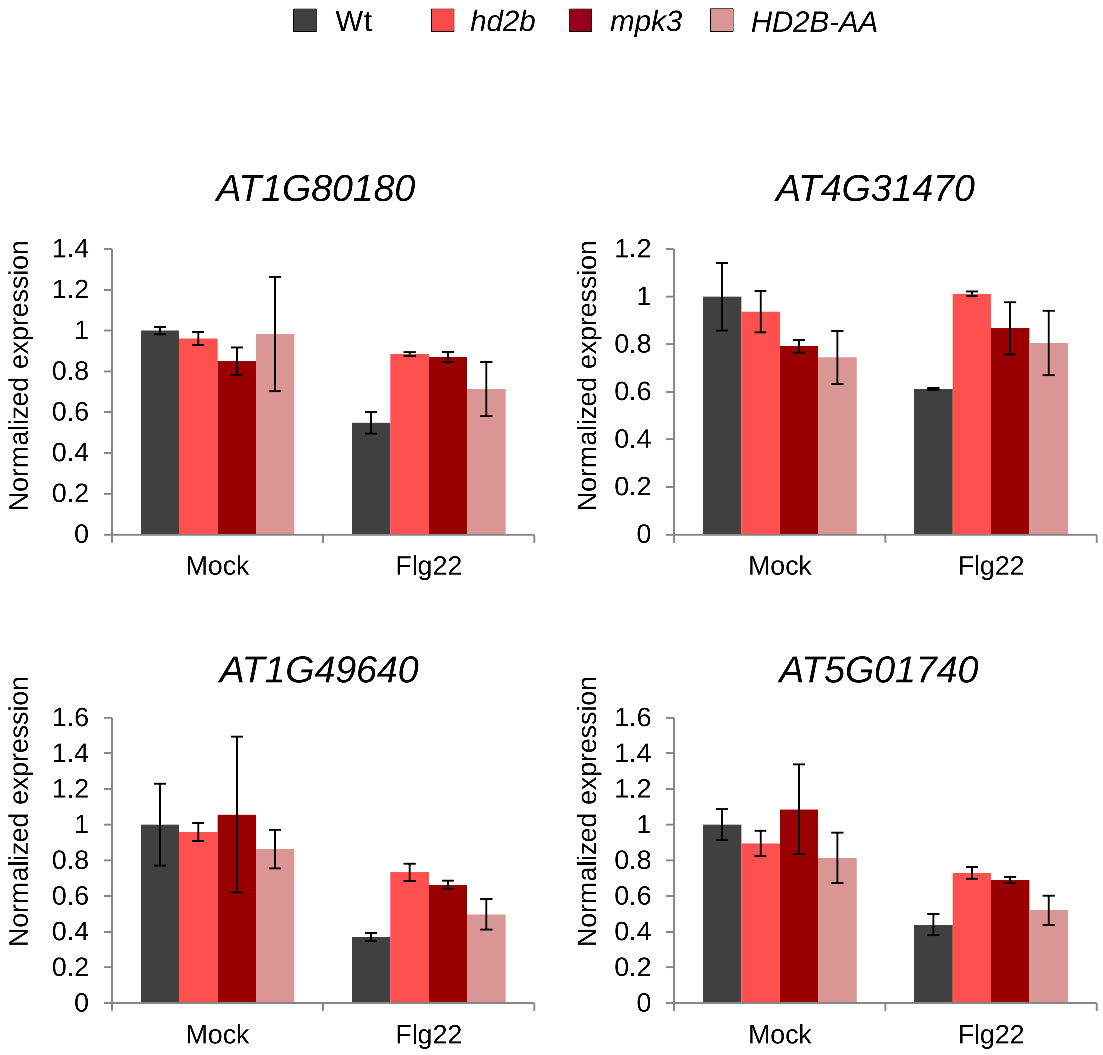


**Figure S28: Gene expression analyses of *hd2b* and *mpk3* flg22 mis-regulated genes in the HD2B-AA line.**

# Additional file 1: Figure S29

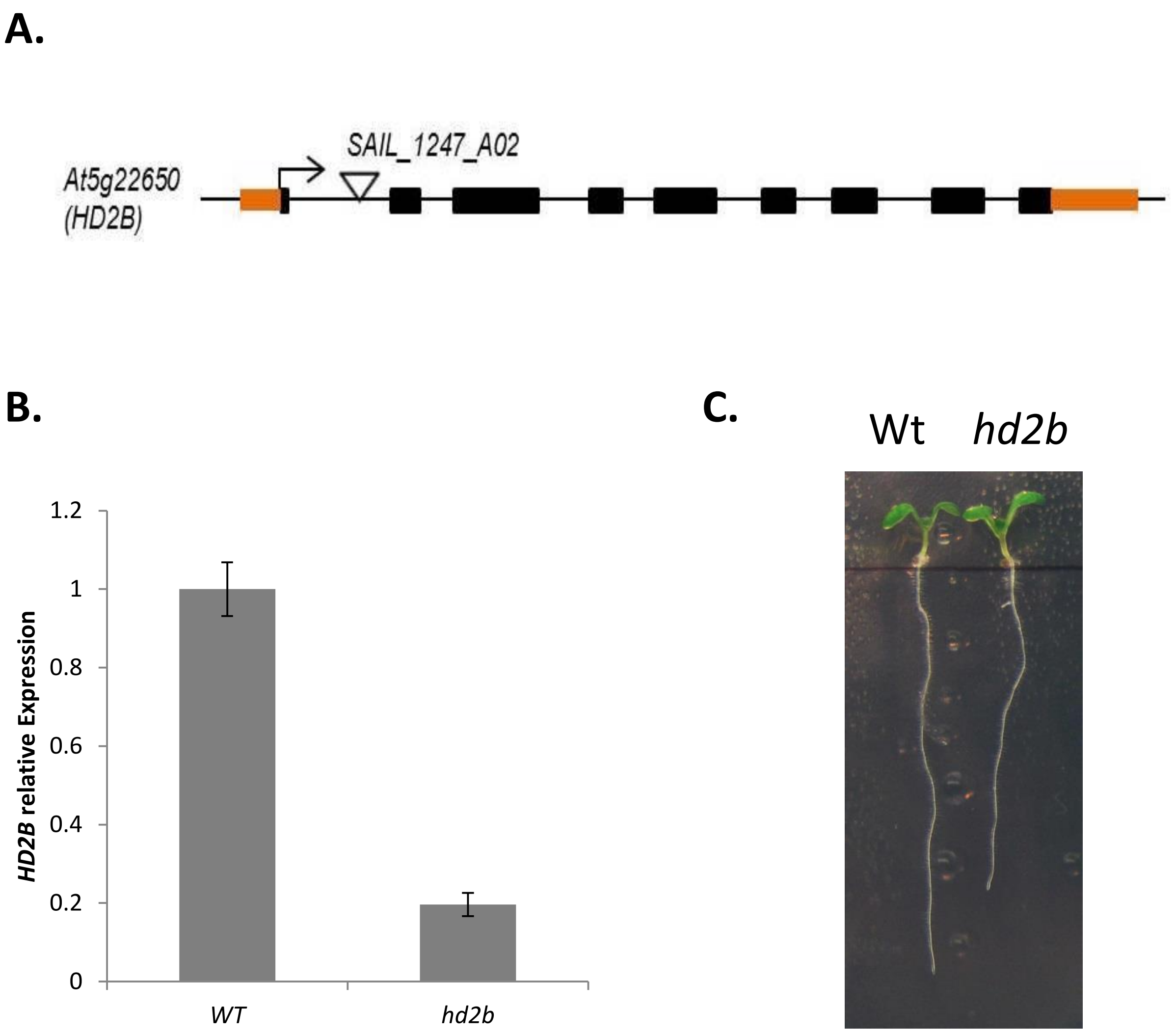


**Figure S29: Characterization of *hd2b* mutant line.**

**A:** Schematic representation of *HD2B* locus indicating the T-DNA insertion position of the *hd2b* mutant.

**B:** RT-qPCR in Wt and *hd2b* T-DNA mutant showing the reduction of HD2B expression in the mutant line.

**C:** Picture of 7-day-old Wt and *hd2b* mutant seedlings. The *hd2b* mutant showed a reduced primary root length but didn’t display any phenotype on shoot.

# Additional file 1: Figure S30

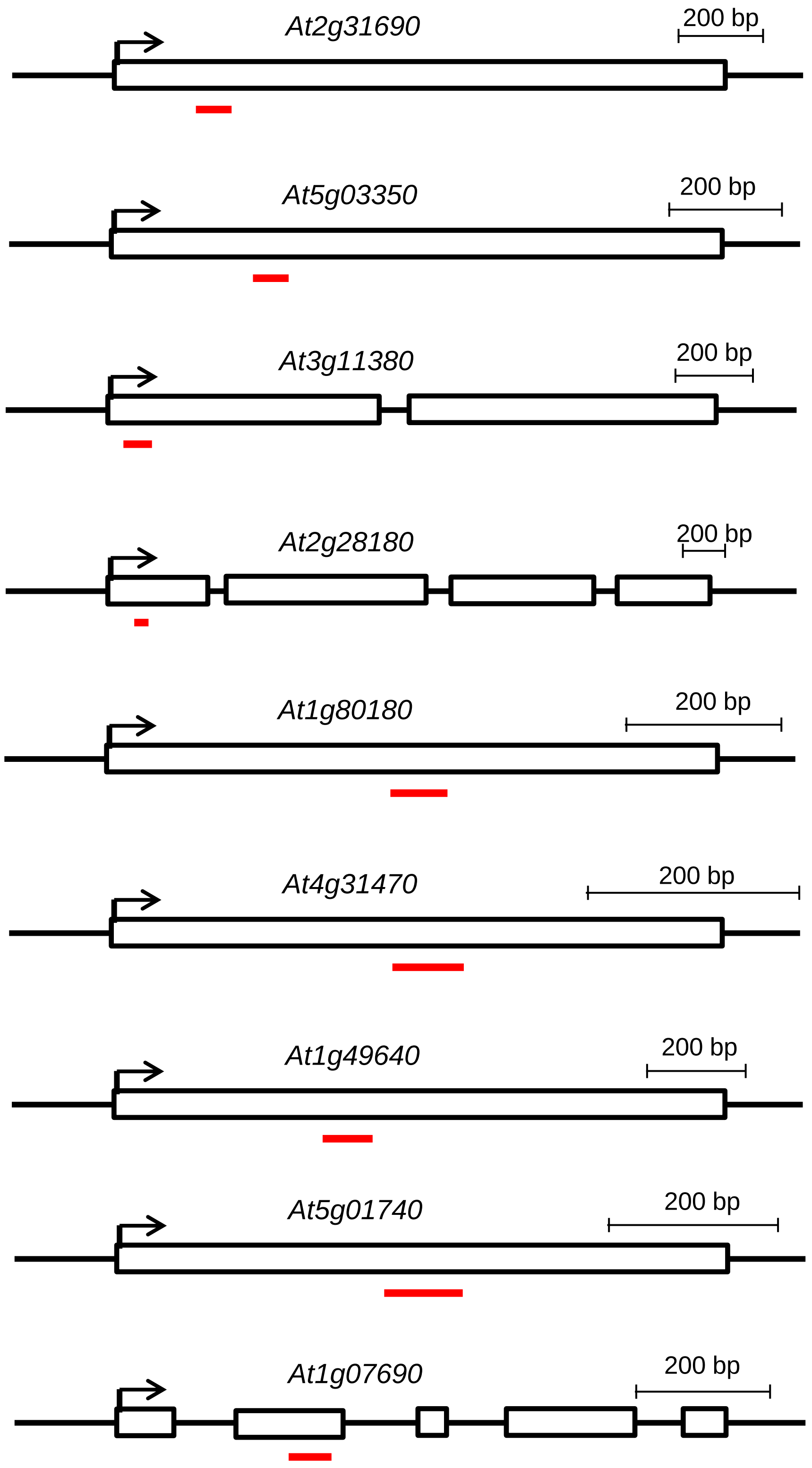


**Figure S30: Positions of the regions analyzed by ChIP-qPCR experiments on the different loci.**
